# RGF signaling bridges root development and nonlethal thermal stress adaptation

**DOI:** 10.1111/nph.71392

**Published:** 2026-07-05

**Authors:** Yu‐Chun Hsiao, Joon‐Keat Lai, Shiau‐Yu Shiue, Masashi Yamada

**Affiliations:** ^1^ Agricultural Biotechnology Research Center Academia Sinica Taipei 115 Taiwan; ^2^ Biotechnology Center in Southern Taiwan Academia Sinica Tainan 711 Taiwan; ^3^ Institute of Tropical Plant Sciences and Microbiology National Cheng Kung University Tainan 701 Taiwan

**Keywords:** *Arabidopsis thaliana*, nonlethal thermal stress, PLT2, reactive oxygen species (ROS), RGF1 signaling, root development, root meristem, thermotolerance

## Abstract

- Roots adapt to growth‐restricting but nonlethal high temperatures. Although lethal heat shock and moderately elevated temperatures have been extensively studied, the effects of nonlethal thermal stress on root development remain unclear. We defined 31°C as a nonlethal thermal stress condition in *Arabidopsis thaliana* and examined its effects using phenotypic and transcriptomic analyses.
- Compared with 22°C, growth at 31°C reduced primary root growth, meristem size, and superoxide (O_2_ ˙^−^) accumulation, accompanied by altered redox states, restricted distribution of the meristem regulator PLETHORA2 (PLT2), and accelerated differentiation. Transcriptome analyses showed repression of meristem‐associated genes and induction of differentiation‐related genes rather than activation of canonical heat‐shock genes.
- Mutants defective in the RGF‐RGFR‐PLT2 pathway were hypersensitive to nonlethal thermal stress. By contrast, exogenous RGF treatment restored primary root meristem defects and promoted lateral root elongation under prolonged stress.
- These findings indicate that the RGF‐RGFR‐PLT2 pathway plays a central role in root adaptation to nonlethal thermal stress and suggest that manipulation of RGF signaling could improve root thermotolerance and crop resilience under elevated temperatures.

## Graphical abstract

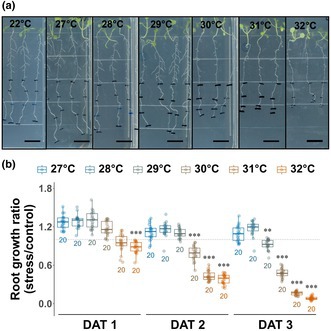

## Introduction

High temperatures induce a range of developmental, physiological, and biochemical changes in plants, affecting plant growth and causing significant reductions in agricultural yields (Lobell *et al*., 2011; Lesk *et al*., 2016). Young seedlings are susceptible to high‐temperature stress, which can cause significant damage to root growth and development, preventing them from accessing soil water and nutrients. Enhancing tolerance to high‐temperature stress in young seedling roots improves both survival rates and yields (Lu *et al*., 2022).

In *Arabidopsis* seedlings, root developmental zones, collectively referred to as zonation, are well characterized. Roots exhibit dynamic growth, tightly coordinated by root zonation, which is divided into the meristematic, elongation, and differentiation zones (Petricka *et al*., 2012). Stem cell daughters undergo active cell divisions in the meristematic zone to produce numerous cells; after that, cells terminate their divisions, rapidly expand in the elongation zone, and finally differentiate to acquire distinct structures in the differentiation zone (Petricka *et al*., 2012). The size of each zone is regulated by environmental signals, phytohormones, small peptides, and endogenous cues such as reactive oxygen species (ROS) (Petricka *et al*., 2012; Hsiao & Yamada, 2020, 2024; Mase & Tsukagoshi, 2021). Because continuous root growth depends on sustained cell division and tightly regulated redox homeostasis in the root apical meristem, this tissue is particularly sensitive to environmental stress. Stress‐induced reductions in meristem size can compromise root growth and function, thereby threatening seedling survival (Ubogoeva *et al*., 2021). These observations suggest that seedlings with larger meristems may exhibit greater resilience under adverse environmental conditions.

ROS were long regarded as harmful byproducts of metabolic stress because their excessive accumulation, particularly under lethal or near‐lethal stress conditions, can damage tissues and trigger cell death (Mittler, 2017). However, under physiological conditions, ROS such as superoxide (O_2_˙^−^) and hydrogen peroxide (H_2_O_2_) also function as spatially regulated signaling molecules. Recent studies have shown that ROS localize to distinct root developmental zones, where their spatial distribution regulates the transition from cell division to elongation. Together, these studies highlight ROS as important regulators of root development (Dunand *et al*., 2007; Tsukagoshi *et al*., 2010; Mittler, 2017; Yamada *et al*., 2020).

Consistent with this developmental framework, ROOT MERISTEM GROWTH FACTOR peptides RGF1, RGF2, and RGF3 (also known as CLE‐like/GOLVEN peptides; hereafter referred to as RGFs) function as key regulators of root meristem maintenance by sustaining meristematic cell proliferation (Matsuzaki *et al*., 2010; Meng *et al*., 2012; Whitford *et al*., 2012). These RGFs act redundantly, and the *rgf1/2/3* triple mutant exhibits a reduced root meristem phenotype that can be rescued by exogenous RGF1 treatment (Matsuzaki *et al*., 2010). RGF1 is perceived by the RGF1 RECEPTORs (RGFRs), also known as RGF1 INSENSITIVEs (RGIs), which are specifically expressed in the meristematic zone (Ou *et al*., 2016; Shinohara *et al*., 2016; Song *et al*., 2016). The RGF‐RGFR signaling pathway therefore represents a key developmental module regulating root meristem maintenance.

We previously showed that exogenous RGF1 elevates O_2_˙^−^ levels in the meristematic zone while reducing H_2_O_2_ in the elongation and differentiation zones in the wild‐type, but not in the *rgfr1/2/3* mutant (Yamada *et al*., 2020). The root meristem master regulator *PLT2* transcript is specifically expressed in the apical root meristem around quiescent center (QC) cells (Galinha *et al*., 2007). By contrast, the PLT2 protein gradient, visualized using the translational fusion line *gPLT2‐YFP*, is detected at high levels around the apical root meristem and gradually decreases toward the end of the meristematic zone (Galinha *et al*., 2007). RGF1‐dependent changes in ROS distribution post‐translationally stabilize the PLT2 protein and enlarge meristem size through expansion of the PLT2 protein gradient (Yamada *et al*., 2020; Hsiao *et al*., 2025). More recently, we further demonstrated that ROS‐dependent regulation of PLT2 stability is mediated through redox‐sensitive cysteine residues within the PLT2 protein (Hsiao *et al*., 2025). Together, these findings established a mechanistic link between RGF signaling, ROS dynamics, and PLT2‐dependent root meristem maintenance.

In addition to RGF1‐3, other RGF members, such as RGF5 and RGF8, are involved in lateral root development (Fernandez *et al*., 2013). The *rgf5/8* double mutant increases total lateral root primordium density as compared with the single mutants and the wild‐type (Fernandez *et al*., 2020). Overexpression of *RGF5* and *RGF8* and treatment with high concentrations of exogenous RGF5 and RGF8 peptides inhibit the formation of the lateral root primordium (Fernandez *et al*., 2015, 2020). The lateral root development in the triple mutant background of the *RGFR1/RGI1*, *RGI4*, and *RGI5* receptor genes, which is not inhibited by *RGF5* overexpression, suggests *RGF5*/*RGF8*‐*RGI* receptor pathways modulate lateral root development (Fernandez *et al*., 2020).

As an environmental signal, high temperatures elicit distinct root responses depending on intensity. Acute heat shock (40–42°C) is lethal to *Arabidopsis* seedlings, but prior exposure to sublethal levels (37°C) induces acclimation through the accumulation of heat‐shock proteins and ROS‐scavenging enzymes (Yeh *et al*., 2012; Ohama *et al*., 2017). By contrast, moderately high temperatures (26–29°C) promote root growth via hormone‐mediated thermomorphogenesis (Quint *et al*., 2016; Vu *et al*., 2019). We can expect that nonlethal thermal stress above 30°C strongly suppresses root growth, limits water and nutrient availability, and ultimately affects whole‐plant development. It is essential to understand the mechanisms underlying acclimation to nonlethal thermal stress and the benefits of this process for improving thermal resilience. However, it remains unclear.

In this study, we define 31°C as a nonlethal yet growth‐restrictive temperature that limits root meristem development. Transcriptome profiling reveals strong repression of *RGF2*, *RGFR2*, and *PLT2* instead of induction of the canonical stress‐response genes, and mutants lacking these genes are particularly sensitive in root meristem and lateral root development to nonlethal thermal stress. Exogenous RGF application counteracts these inhibitory effects of root meristem development and lateral root growth. Together, these findings demonstrate that RGF peptide signaling plays a crucial role in root adaptation to nonlethal thermal stress by modulating established root developmental programs rather than stimulating the canonical heat‐shock responses at elevated temperatures. Our findings also highlight the potential of RGF peptide application as a strategy to counteract heat‐induced root growth inhibition and to strengthen the resilience of root architecture in warming environments.

## Materials and Methods

### Plant material and growth conditions

All *Arabidopsis thaliana* (L.) Heynh. mutants and transgenic lines used in this study (SALK_130119.20.25 (*plt2*), *rgf123*, *rgfr123, gPLT2‐YFP*, *cytGRX*‐*roGFP2*, and *CycB1;1‐GFP*) are in the Columbia‐0 (Col‐0) genetic background and have been previously characterized (Galinha *et al*., 2007; Meyer *et al*., 2007; Ubeda‐Tomas *et al*., 2009; Matsuzaki *et al*., 2010; Shinohara *et al*., 2016; Yamada *et al*., 2020; Hsiao *et al*., 2025). Seeds were surface‐sterilized with 50% bleach supplemented with 0.1% Tween 20 (Sigma) for 15 min, followed by five rinses with sterile distilled water. The sterilized seeds were then stratified at 4°C in the dark for 2 d. Subsequently, seeds were sown on half‐strength Murashige and Skoog (½ MS) medium (Caisson Laboratories: Smithfield, UT, USA) supplemented with 0.05% MES, 1% sucrose, and 1% agar (Sigma) and pH adjusted to 5.7 with KOH. Seedlings were grown vertically in a growth chamber (F‐600 HiPoint, Taiwan) at 22°C under long‐day conditions (16 h : 8 h light : dark) for 7 d. After this period, seedlings were transferred to ½ MS agar plates containing either sterile water (mock treatment), synthetic sulfated RGF1 peptide (Mission Biotech, Taipei, Taiwan), or subjected to the indicated temperature treatments for 1–3 d for further experiments.

### Root growth assay

Seven‐day‐old Arabidopsis seedlings were transferred to growth chambers set at different temperatures (22, 27–32°C) for 3 d. Primary root growth was recorded daily at the same time each day, with root tip positions marked daily. After the final observation on day three, images of the plates were scanned using an Epson Perfection V800, and root lengths were measured using Fiji ImageJ (Schindelin *et al*., 2012) and normalized to those of seedlings grown at 22°C.

For lateral root observation, 7‐d‐old Arabidopsis seedlings were transferred to the indicated media and grown at 22°C or 31°C for 6 d. Root images were captured using the same scanner, and lateral root lengths were measured with Fiji ImageJ (Schindelin *et al*., 2012). The average lateral root length per seedling was calculated and used for statistical analysis.

### Light and confocal microscopy analysis

For superoxide (O_2_˙^−^) detection, seedlings were stained with 400 μM nitroblue tetrazolium (NBT) dissolved in 20 mM phosphate buffer (pH 6.1) in the dark for 2 min, followed by two rinses with distilled water, as previously described (Hsiao *et al*., 2025). Root images were captured using a 10× objective lens on a Zeiss AXIO Scope A1 microscope.

For quantitative analysis of NBT staining, signal intensity was measured specifically within the anatomically defined meristematic zone, as described below. Total NBT signal intensity within this region was quantified using Fiji ImageJ and used for statistical analysis. Because meristem size itself is a biologically meaningful output of nonlethal heat stress, NBT signals were intentionally not normalized to meristem area.

Root tip structures and fluorescent signals were observed using a 20× objective on a Zeiss LSM 980 laser‐scanning confocal microscope. Excitation and emission settings were as follows: YFP, excitation at 514 nm and emission at 517–544 nm; GFP, excitation at 488 nm and detection at 491–544 nm; propidium iodide staining, excitation at 561 nm and emission at 565–747 nm.

Confocal images were stitched using the method described by (Thevenaz & Unser, 2007), and the number of cortex cells in the meristematic zone was quantified. The meristematic zone was defined as the region extending from the QC to the first cortex cell whose longitudinal (*y*‐axis) length exceeded twice that of the preceding cell. The number of cortex cells within this region was counted to estimate meristem size.

### Confocal imaging and redox‐state analysis of the GRX‐roGFP2 biosensor

Seedlings expressing *cytGRX‐roGFP2* (Meyer *et al*., 2007) were grown on half‐strength Murashige and Skoog (½ MS) agar plates for 7 d at 22°C in a growth chamber. Seedlings were then transferred to either 22°C or 31°C and treated with mock or 5 nM RGF1, followed by propidium iodide (PI) staining. Fluorescence signals from roGFP2 and PI were acquired using a Zeiss LSM 980 laser‐scanning confocal microscope. Images were collected as single optical sections. For roGFP2 imaging, excitation was performed at 405 and 488 nm, and emission was collected at 505–529 nm. For PI imaging, excitation was performed at 561 nm, and emission was collected at 565–600 nm. Single‐plane images were obtained using the QC and cortex cells as positional landmarks, and optical sections were acquired along the cortex cell layer from the root tip to the differentiation zone. Because signal intensity in single optical sections differed markedly between inner and outer tissues, quantitative analysis was restricted to anatomically defined regions near the central cortex, excluding epidermal, lateral root cap, and columella cells.

Reduced–oxidized (RO) index images were generated using a custom redox‐state analysis pipeline as described in Supporting Information Notes S1. Briefly, the region of interest in the image was obtained by image segmentation, and the RO index was subsequently computed with the standardization and normalization process between the 405 and 488 nm channels. Regions exhibiting relatively oxidized or reduced states were distinguished based on the sign of the RO index. The distance from the QC to the first detectable oxidized cell domain was measured as an indicator of spatial redox‐state shift. In addition, the mean RO index values were quantified in regions proximal to the oxidized cell domain or within the meristematic zone using Fiji/ImageJ (Schindelin *et al*., 2012).

### Real‐time qPCR analysis

Total RNA concentration was quantified using a Qubit fluorometer (Invitrogen). cDNA synthesis was performed using SuperScript IV Reverse Transcriptase (Invitrogen). qPCR was conducted using SYBR Green Supermix (Bio‐Rad) on a CFX Connect Real‐Time PCR Detection System (Bio‐Rad). Transcript levels were normalized to *PP2AA3* (At1g13320). Three biological replicates were analyzed for each experiment. Primer sequences used in this study are listed in Dataset S1.

### RNA‐seq library preparation and data processing

Similarly sized root tip tissues encompassing the meristematic, elongation, and differentiation zones were precisely excised on a 2% agarose plate containing ½ MS using an ophthalmic scalpel (Feather: Osaka, Japan) under a dissection microscope (Leica M205 FCA). The dissected region extended *c*. 1000–1100 μm from the QC, thereby encompassing the three major developmental zones of the root tip while excluding most mature root tissues and enriching for developmental‐zone‐specific transcriptional responses. The dissected root tip tissues were then homogenized in RNA extraction buffer and processed according to the manufacturer's recommended protocol. RNA extracted from 50 root tissues was pooled for each sample. Total RNA was extracted using the RNeasy Micro Kit (Qiagen) according to the manufacturer's instructions. RNA concentration was quantified using a Qubit fluorometer (Invitrogen). RNA quality was assessed using a 2100 Bioanalyzer (Agilent: Santa Clara, CA, USA), and all samples exhibited RNA integrity numbers above 8.0. RNA‐seq libraries were prepared from 300 ng of total RNA using the Universal Plus Total RNA‐Seq with NuQuant Library Preparation Kit (Tecan Trading AG: Männedorf, Switzerland), following the manufacturer's instructions. Libraries from five biological replicates of root samples under the control temperature (22°C) and nonlethal high temperature (31°C) at 1 and 2 d after transfer (DAT) were sequenced on an Illumina NextSeq 3300 platform, generating 150‐base paired‐end reads. The RNA‐sequencing reads were aligned to the Arabidopsis reference genome TAIR10 using subread (Liao *et al*., 2013). The mRNA read counts of genes were computed using featureCounts (Liao *et al*., 2014). Genes expressed (read counts > 0) are included for analysis. Normalization for sequencing depth and differential gene analysis was performed using DESeq2 (Love *et al*., 2014). Genes with FDR < 5% were considered differentially expressed. Gene ontology (GO) analysis was performed using the Bioconductor package clusterProfiler (Xu *et al*., 2024). All DEGs can be found in Dataset S2.

### Statistical analysis and reproducibility

Experiments were independently repeated three times with similar results. No power analysis was done to estimate the sample size. All statistical analyses were performed using R v.4.5.0 (http://www.r‐project.org/). For two‐sample comparison, data were analyzed with a two‐tailed *t*‐test. As an omnibus test, an analysis of variance (ANOVA) or an aligned rank transform (ART)‐ANOVA was performed, depending on the data normality. As a *post hoc* test, ART‐contrast test was applied to nonnormal data, and the Games–Howell test, Tukey's tests, or Ryan‐Einot‐Gabriel‐Welsch Q (REGWQ) test was performed on normal data, depending on the data homoscedasticity and sample sizes. In the boxplots, the circles represent each individual data point; the horizontal line within boxes represents the median; the upper and lower hinges, respectively, indicate the 75th and 25th percentiles (interquartile range, IQR); and the whiskers show the 1.5 extension of the IQR.

## Results

### Temperatures between 30°C and 32°C restrict root growth, reduce meristematic cell proliferation, and suppress O_2_ ˙^−^ accumulation

**Fig. 1:**
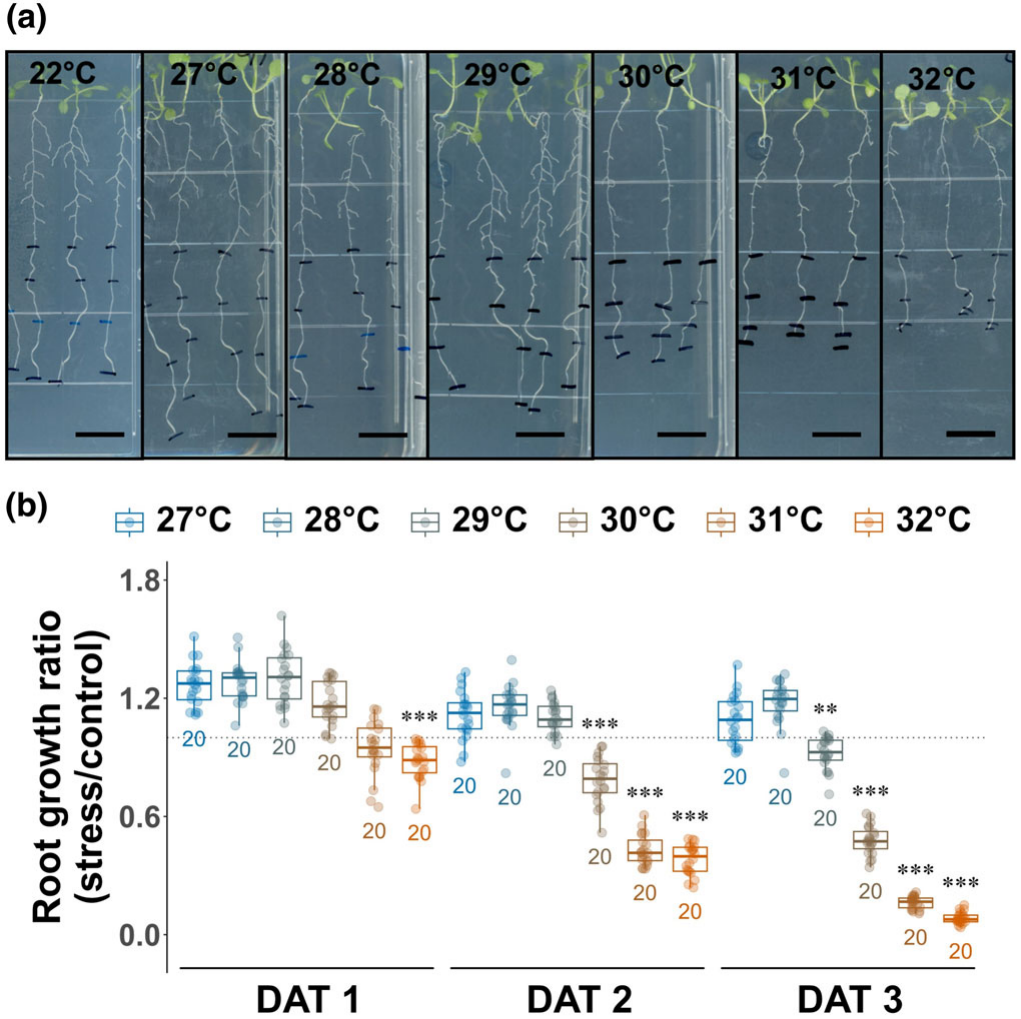
Nonlethal high temperature inhibits root growth in *Arabidopsis thaliana*. (a) Primary root lengths were recorded daily for 3 d after transfer (DAT) to control (22°C) or to elevated temperatures (27–32°C). The topmost black line in each image indicates the root tip position at 0 DAT marked on the Petri dish lid. The locations of root tips were subsequently marked every 24 h. Bars, 10 mm. (b) Root growth ratios were calculated as the relative root length under thermal stress (27–32°C) compared with the control condition (stress/control) at each of the 3 DAT. The average root length measured at 22°C was defined as 1. Asterisks indicate where the root growth ratio line marks the ratio of the heat treatment group is significantly less than the control (*t*‐test or Wilcoxon rank sum test, depending on the data normality; ***, *P* < 0.001; **, *P* < 0.01). Integer values below the boxplots represent the sample size of each group. Gray‐dotted value of 1. In the boxplots, the horizontal represents the median; the upper and lower hinges indicate the 75^th^ and 25^th^ percentiles (interquartile range, IQR); whiskers extend to 1.5 × the IQR; and circles represent individual data points.

Although elevated temperatures are known to inhibit root growth, the threshold and underlying developmental responses remain unclear. We first wished to identify the threshold temperature at which root growth is impaired. To do so, Arabidopsis seedlings were grown at 22°C for 7 d and then transferred to either the control condition (22°C) or elevated temperatures (27, 28, 29, 30, 31, or 32°C). Root length was measured every 24 h for 3 d, and growth ratios were calculated relative to 22°C. At 1 DAT, seedlings transferred to 32°C showed a slight but significant reduction in root growth, whereas the growth ratios of seedlings transferred to the other temperatures were not significantly different from those maintained at 22°C (Fig. 1a,b). By 2 DAT, however, growth ratios of plants transferred to 30, 31, and 32°C declined. By 3 DAT, root growth was modestly reduced in seedlings transferred to 29°C, strongly suppressed at 30–31°C, and nearly abolished at 32°C (Fig. 1a,b).

These results indicate that root growth inhibition occurs progressively across a temperature range between 30 and 32°C, rather than at a single discrete threshold. We therefore selected 31°C as a representative nonlethal, growth‐restrictive condition in which roots remain viable and continue to elongate, providing a practical experimental window for quantitative analysis of developmental and molecular responses to elevated temperature.

**Fig. 2:**
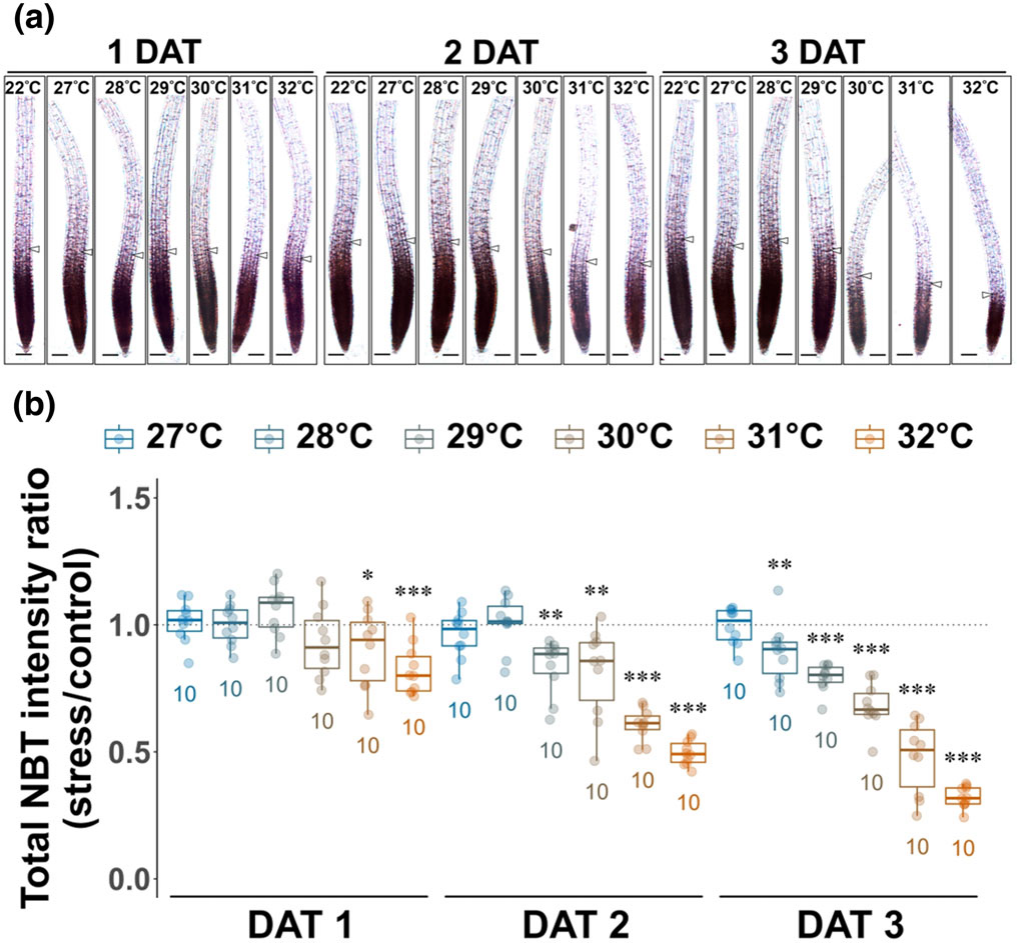
Nonlethal thermal stress decreases O_2_˙^−^ levels in the *Arabidopsis thaliana* root meristem. (a) Light microscopy images of Col‐0 root tips stained with nitroblue tetrazolium (NBT) at 1, 2, or 3 d after transfer (DAT) to either control conditions (22°C) or elevated temperatures (27–32°C). Images were captured under bright‐field illumination at 1× magnification. Representative images are shown; quantitative conclusions are based on statistical analyses of total NBT signal intensity across multiple biological replicates rather than on individual images. Representative pictures shown; *n* = 3 independent experiments were performed. Each experiment included more than 15 roots. Arrowheads indicate the junction between the meristematic and elongation zones (see the ‘Materials and Methods’ section). Bars, 100 μm. (b) Quantitative analysis of NBT staining in Col‐0 roots under the indicated conditions. NBT signals were quantified as the total staining intensity within the anatomically defined meristematic zone, rather than as area‐normalized intensity. Because meristem size itself represents a biologically meaningful output of nonlethal heat stress, total NBT signal intensity was used to capture combined changes in meristem structure and superoxide (O_2_˙^−^) distribution. The intensity of the control group (22°C) was set to 1. Asterisks indicate that the NBT total intensity ratio of the heat treatment group is significantly less than the control (*t*‐test or Wilcoxon rank sum test, depending on the data normality; ***, *P* < 0.001; **, *P* < 0.01; *, *P* < 0.05). The integer values below the boxplots represent the sample sizes of each group. The gray‐dotted line marks the ratio value of 1. In the boxplots, the horizontal line represents the median; the upper and lower hinges indicate the 75^th^ and 25^th^ percentiles (interquartile range, IQR); whiskers extend to 1.5 × the IQR; and circles represent individual data points.

**Fig. 3:**
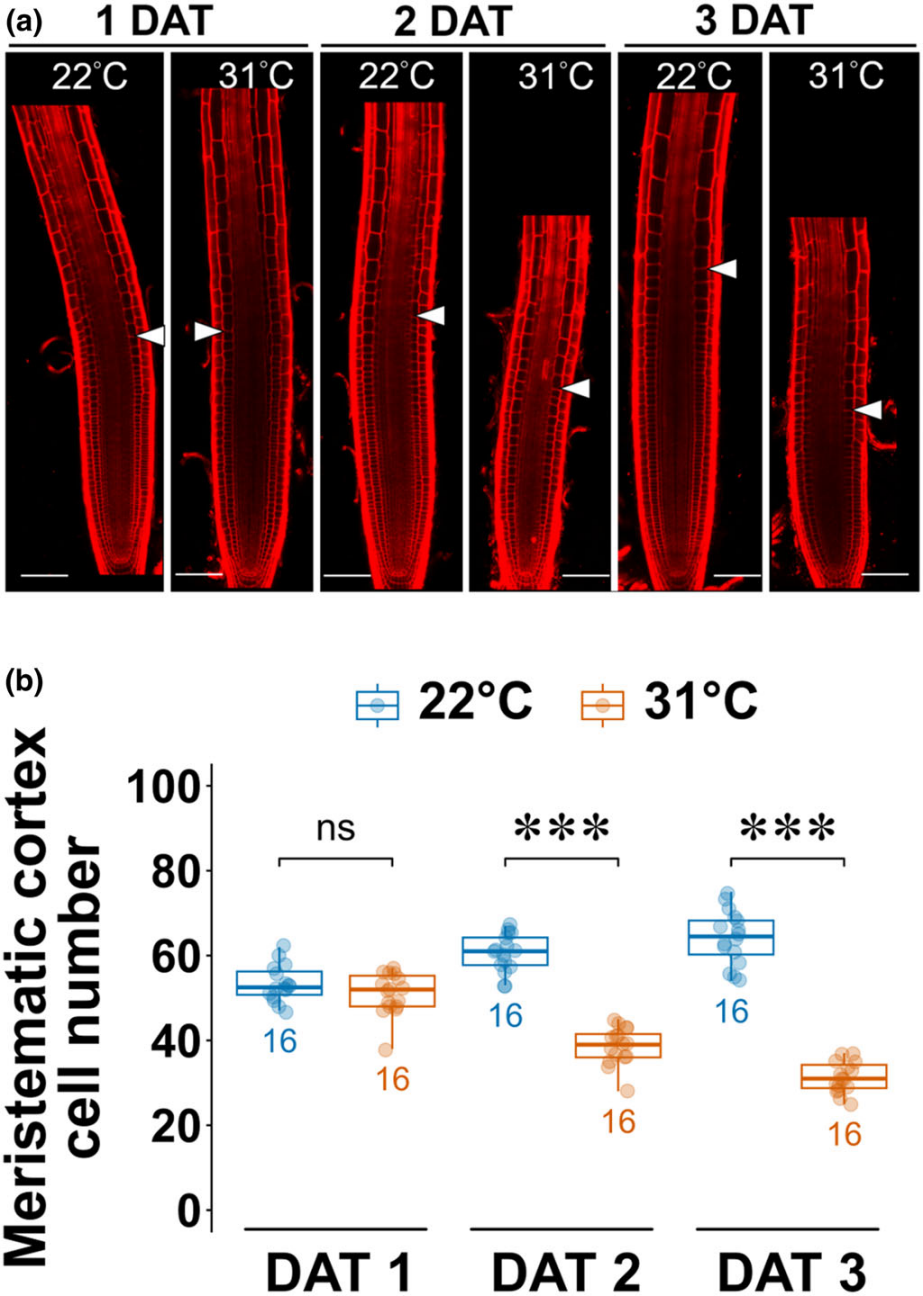
The meristematic zone size decreases during adaptation to nonlethal thermal stress in *Arabidopsis thaliana*. (a) Confocal microscopy was used to capture daily images of the three developmental zones for 3 d after transfer (DAT) to control (22°C) and heat‐stress temperature (31°C) conditions. Arrowheads indicate the boundary between the meristematic zone and the elongation zone, defined according to the established criterion based on cortical cell length, as the region between the quiescent center and the last cortical cell that had not doubled in length relative to its predecessor (see the ‘Materials and Methods’ section). Representative images are shown; *n* = 3 independent experiments. Each experiment included more than 15 roots. Bars, 100 μm. (b) Quantitative analysis of meristematic cortex cell number in roots grown under the indicated conditions. Meristem size was quantified by counting cortical cell number rather than by area measurement. Asterisks indicate that the meristematic cortex cell of the heat treatment group is significantly different from the control (*t*‐test; ***, *P* < 0.001), while ns indicates nonsignificant differences (*P* > 0.05). In the boxplots, the circles represent each individual data point, and sample sizes are shown below the corresponding boxes. The horizontal line within the boxes represents the median, and the upper and lower hinges, respectively, indicate the 75^th^ and 25^th^ percentiles (interquartile range, IQR). The whiskers extend to 1.5 × the IQR.

Spatial distributions of ROS in the root developmental zones modulate root growth (Dunand *et al*., 2007; Tsukagoshi *et al*., 2010). O_2_˙^−^ preferentially accumulates in the meristematic zone, in correlation with root length and meristematic zone size (Tsukagoshi *et al*., 2010; Yamada *et al*., 2020). We therefore hypothesized that O_2_˙^−^ decreases concomitantly with root growth inhibition under elevated temperatures. To test this, O_2_˙^−^ levels were assessed by nitroblue tetrazolium (NBT) staining. Staining intensity in the meristematic zone decreased in seedlings transferred to warmer temperatures at 2 DAT and was markedly reduced by 3 DAT at 31–32°C compared with 22°C (Fig. 2a,b). Thus, root growth inhibition (Fig. 1) and reduced O_2_˙^−^ accumulation (Fig. 2) occurred in parallel at 31–32°C. We noted that, while root growth was almost completely arrested at 32°C, roots at 31°C continued to elongate slowly (Fig. 1a), indicating that 31°C provides a nonlethal condition suitable for studying root adaptation. Concurrent reductions of root growth and O_2_˙^−^ accumulation suggest downsizing of the meristematic zone under nonlethal thermal stress (31°C). To test this, we measured cell number in the meristematic zone as tracked meristem zone size using confocal imaging under nonlethal thermal stress at the same time points. We found that at 1 DAT, meristematic zone size was similar in plants that had been transferred to 31 and 22°C (Fig. 3a,b). By 2–3 DAT, however, the cell number in the meristematic zone was significantly reduced at 31°C (Fig. 3a,b), consistent with the reduced root growth and O_2_˙^−^ accumulation we observed in Figs 1, 2. To further assess meristematic cell proliferation at 2 DAT, when meristem size reduction became apparent, we analyzed the mitotic cell‐cycle marker CYCB1;1‐GFP (Ubeda‐Tomas *et al*., 2009) under nonlethal thermal stress conditions. At 31°C, the CYCB1;1‐GFP signal was detected within a substantially narrower region than at 22°C at 2 DAT (Fig. S1a,b), indicating contraction of the actively proliferating meristematic domain. Consistent with this observation, the meristematic zone in the same roots was also smaller at 31°C than at 22°C (Fig. S1c).

Despite the reduction in meristematic zone size, no typical morphological hallmarks of cellular damage were detected in the apical root meristem under our experimental conditions (Fig. 3a). Rather than showing typical cellular damage, premature cell elongation and differentiation were observed at 31°C compared with 22°C (Fig. 3a, arrowheads show the junction between the meristematic zone and elongation zone). Together, these findings show that nonlethal thermal stress of 31°C restricts root meristem development and accelerates cell differentiation through a coordinated developmental mechanism. We attempted to assess H_2_O_2_ distribution under these conditions using the H_2_O_2_‐specific probe H_2_O_2_‐BES‐Ac (Maeda *et al*., 2004; Yamada *et al*., 2020; Hsiao *et al*., 2025); however, the probe showed poor penetration and unstable staining under nonlethal thermal stress conditions, presumably because accelerated differentiation promoted the formation of apoplastic barriers such as the Casparian strip, thereby preventing reliable detection of H_2_O_2_.

We therefore focused on O_2_˙^−^ dynamics, which are closely associated with meristem activity and root developmental regulation. O_2_˙^−^ distributions were nevertheless reduced at 31°C, suggesting that nonlethal thermal stress alters the redox state in specific root developmental zones.

Having established 31°C as a nonlethal thermal stress condition that restricts meristem growth, meristematic cell proliferation, and reduces O_2_˙^−^ accumulation, we next asked how these changes are reflected in the broader cellular redox landscape. Although reduced O_2_˙^−^ accumulation suggested altered redox status under nonlethal thermal stress, other ROS species such as H_2_O_2_ may also contribute to these changes. However, reliable detection of H_2_O_2_ using H_2_O_2_‐BES‐Ac was not possible under our experimental conditions because the probe showed poor penetration and unstable staining at elevated temperatures. Therefore, we employed the genetically encoded redox sensor GRX‐roGFP2 (Meyer *et al*., 2007) to monitor the intracellular glutathione‐dependent redox status. This approach enabled assessment of the overall intracellular redox environment rather than individual ROS species alone.

### Root development adapts to nonlethal thermal stress by altering redox states in specific developmental zones

**Fig. 4:**
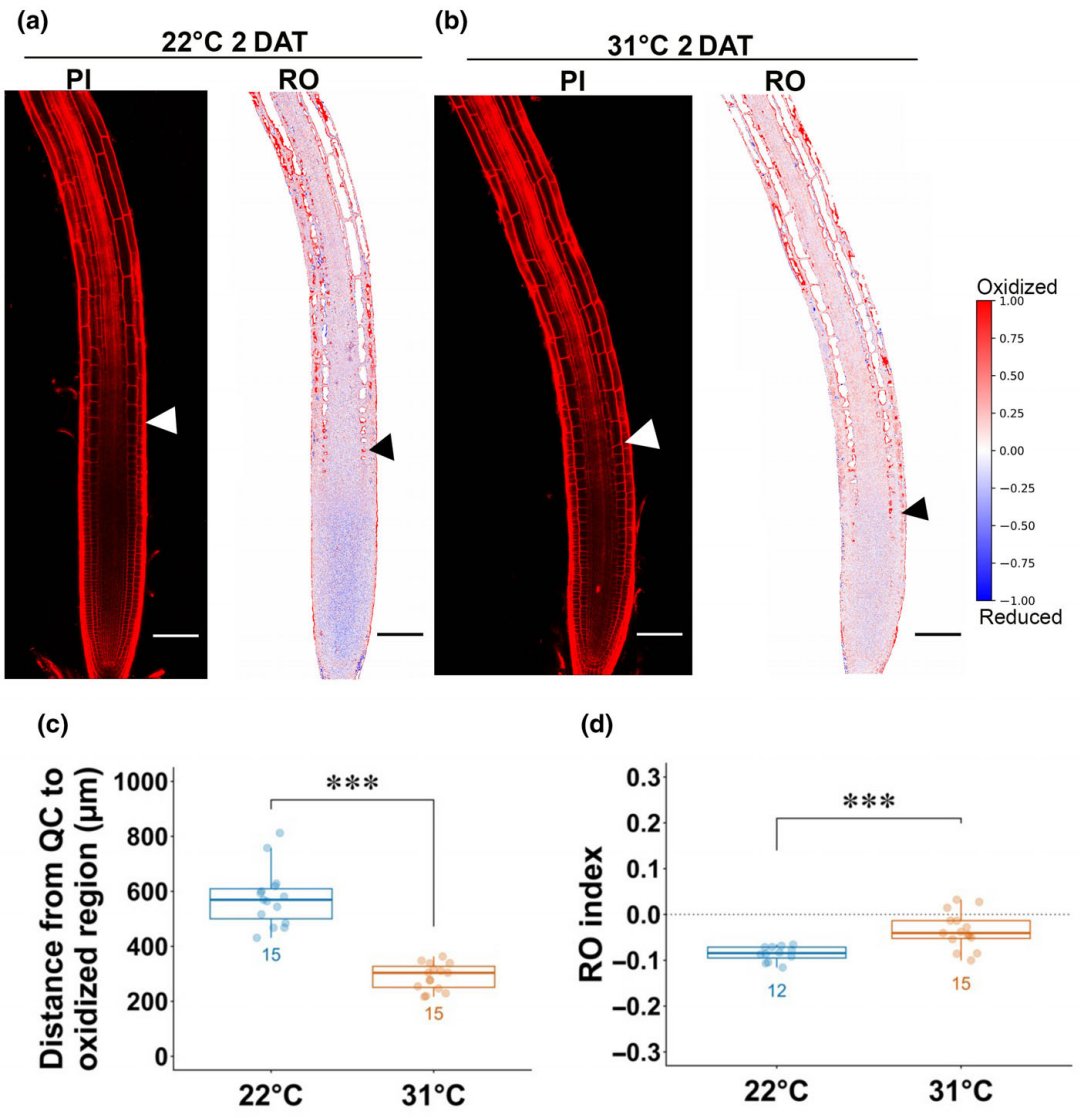
Nonlethal thermal stress repositions redox domains during root developmental progression in *Arabidopsis* *thaliana*. (a, b) Representative single‐plane confocal images of cytosolic GRX‐roGFP2 (cytGRX‐roGFP2) in Arabidopsis roots at 2 d after transfer (2 DAT) under control (22°C) and nonlethal heat stress (31°C) conditions. Images were acquired at a single optical section to visualize cortex cells. PI staining marks cells (left panels at each temperature). Reduced–Oxidized (RO) index represents relative cellular redox status (right panels at each temperature). White arrowheads indicate the boundary between the meristematic zone and elongation zone. Black arrowheads indicate the first detectable oxidized cell domain. Bars, 100 μm. (c,d) Quantitative analysis of (a) and (b). (c) Distance from the QC to the first detectable oxidized cell domain as an indicator of spatial redox‐state shift. (d) Mean RO index values quantified in regions proximal to the oxidized cell domain within the meristematic zone. Asterisks indicate significant differences determined by Student's *t*‐test (***, *P* < 0.001). In the boxplots, the circles represent each individual data point, and sample sizes are shown below the corresponding boxes. The horizontal line within boxes represents the median, and the upper and lower hinges, respectively, indicate the 75^th^ and 25^th^ percentiles (interquartile range, IQR). The whiskers extend to 1.5 × the IQR.

To examine how redox states are distributed along the root developmental axis, we analyzed cytosolic GRX‐roGFP2 (Meyer *et al*., 2007) signals and quantified a reduced–oxidized (RO) index at 2 DAT under 22 and 31°C conditions (Fig. 4). PI was used as a counterstain to visualize cell structures.

We observed that the boundary (white triangles) between the meristematic and elongation zones was positioned closer to the root tip under nonlethal thermal stress compared with control conditions, consistent with the phenotype observed in wild‐type plants (Figs 3, 4a,b). Under 22°C, regions with a higher RO index (Fig. 4a, red signals) were detected in elongated and differentiated cells within the elongation and differentiation zones, as well as in the lateral root cap. These observations are consistent with previously reported developmental zone‐specific ROS patterns (Yamada *et al*., 2020). In the cortex layer, cells with a higher RO index began to appear just proximal to the elongation zone at 22°C (Fig. 4a, right image, black triangle). By contrast, under 31°C conditions, cells with a higher RO index appeared closer to the root tip (Fig. 4b, right image, black triangle). Furthermore, elongated cells appeared prematurely under nonlethal thermal stress (Fig. 4b, white triangle).

To quantify this shift, we measured the distance from the QC to the first detectable cells with a higher RO index (Fig. 4c). This distance was significantly shorter under nonlethal thermal stress than under control conditions (Fig. 4c), indicating that oxidation‐associated signals and developmental zone transitions occur closer to the root tip under elevated temperature.

We next quantified the mean RO index in the meristematic zone located proximal to the first detectable cells with a higher RO index. Under control conditions (22°C), this region exhibited a lower RO index and a more reduced state, whereas under nonlethal thermal stress (31°C), it exhibited a higher RO index and a more oxidized state (Fig. 4d). These observations are consistent with the reduced NBT staining observed under nonlethal thermal stress (Fig. 2).

Together, these results indicate that the positioning of redox domains is tightly coupled with developmental zone progression under nonlethal thermal stress. We next investigated the molecular mechanisms underlying this adaptive response.

### Nonlethal thermal stress modulates the RGF peptide‐RGF receptor‐PLT2 signaling pathway

Most transcriptome studies in Arabidopsis have analyzed whole seedlings or entire roots exposed to lethal or moderately high temperatures (Larkindale & Vierling, 2008; Quint *et al*., 2016; Vu *et al*., 2019; Guo *et al*., 2022). While these studies have provided important global insights, they lack spatial resolution and do not address nonlethal thermal stress. More recently, single‐cell RNA‐seq under lethal heat stress (Jean‐Baptiste *et al*., 2019) has enabled cell‐type‐specific profiling; however, zone‐specific responses to nonlethal temperatures remain poorly understood.

**Fig. 5:**
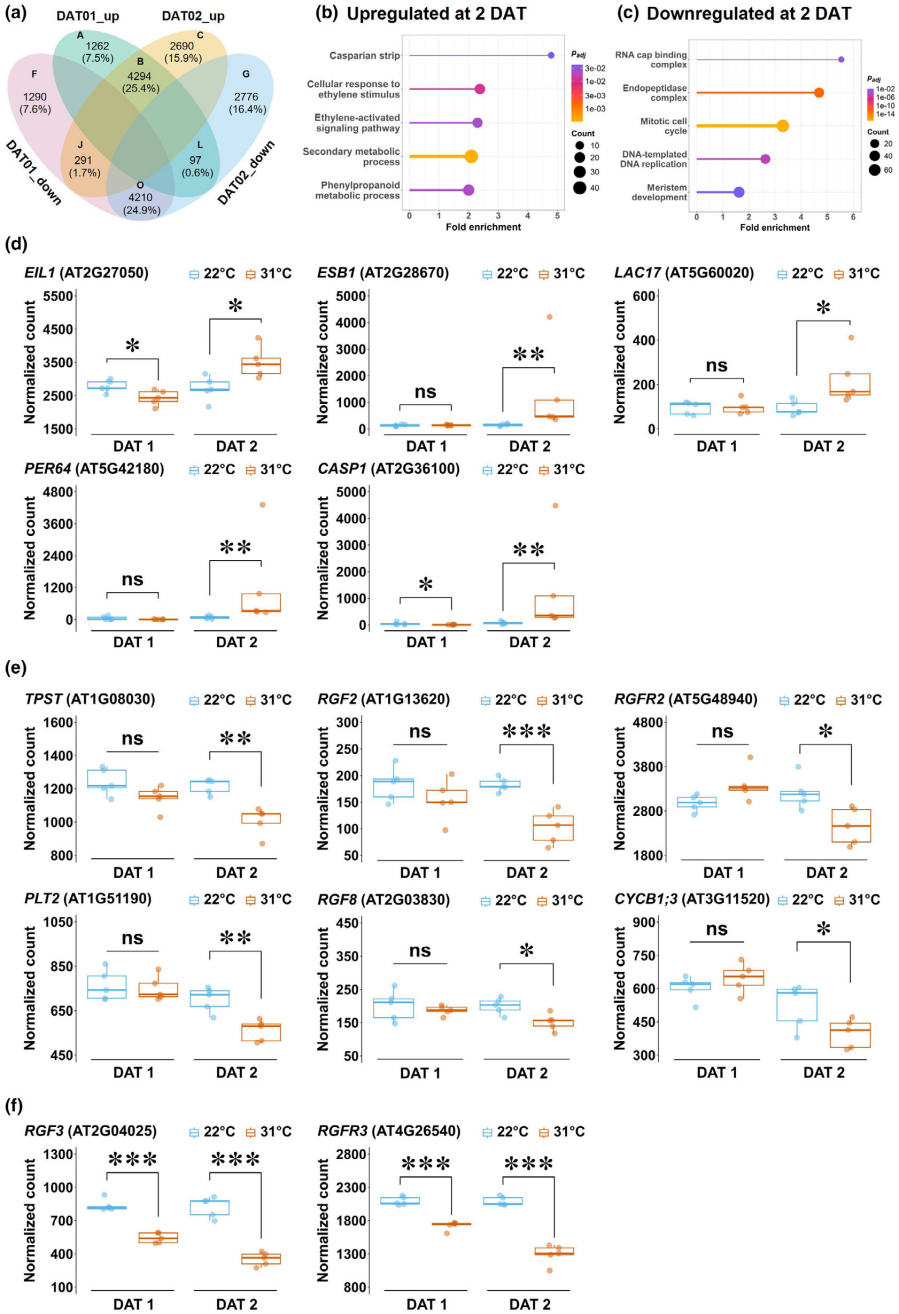
Nonlethal thermal stress represses meristem‐associated genes and promotes elongation and differentiation programs in *Arabidopsis thaliana*. (a) A four‐set Venn diagram illustrating the number of differentially expressed genes (DEGs) under the nonlethal thermal condition (31°C) compared with the control condition (22°C). Upregulated genes at 1 d after transfer (DAT) and/or 2 DAT are shown in the green and orange ellipses, respectively. Downregulated genes at 1 DAT and/or 2 DAT are shown in the red and blue ellipses, respectively. Each DEG subset is designated by a capital letter. The integer values indicate the number of DEGs in each subset, and the percentages represent the proportion of DEGs in each subset relative to the total number of DEGs. (b, c) Gene Ontology (GO) analysis of upregulated (b) and downregulated (c) genes after 2 d of nonlethal heat treatment (subsets C and G, respectively). (d) Cell elongation‐ and endodermal differentiation‐related genes from subset C that are specifically upregulated at 2 DAT. (e) RGF1‐receptor‐PLT2 pathway‐related genes from subset G that are downregulated only at 2 DAT. (f) RGF1‐receptor‐PLT2 pathway‐related genes from subset O that are downregulated at both 1 and 2 DAT. Asterisks indicate significant differences in gene expression between the heat‐treated and control groups (*t*‐test; ***, *P* < 0.001; **, *P* < 0.01; *, *P* < 0.05), whereas ns indicates no significant difference (*P* > 0.05). In the boxplots, circles represent individual biological replicates, and sample sizes are shown below the corresponding boxes. The horizontal line within each box represents the median, and the upper and lower hinges indicate the 75^th^ and 25^th^ percentiles (interquartile range, IQR), respectively. The whiskers extend to 1.5 × the IQR.

To address this gap, we performed transcriptome analyses of three root developmental zones under nonlethal thermal stress (31°C). Total RNA was collected from seedlings at 1 and 2 d DAT to either 22 or 31°C, followed by comparative analyses. We identified > 1200 differentially expressed genes (DEGs) at 1 DAT, > 2600 DEGs at 2 DAT, and *c*. 4200 DEGs across both time points (Fig. 5a).

Consistent with the reduction in meristem size and earlier cell elongation at 31°C, particularly at 2 DAT (Figs 3, 4), GO enrichment analyses revealed significant upregulation of genes associated with cell elongation, secondary metabolism, and endodermal differentiation processes, including Casparian strip formation (Fig. 5b), whereas genes associated with meristem development, the mitotic cell cycle, and DNA replication were significantly downregulated (Fig. 5c).

Notably, genes associated with differentiation‐related processes, including *ENHANCED SUBERIN 1* (*ESB1*), *LACCASE 17* (*LAC17*), *PEROXIDASE 64* (*PER64*), and *CASPARIAN STRIP MEMBRANE DOMAIN PROTEIN 1* (*CASP1*), were significantly upregulated at 31°C at 2 DAT (Fig. 5d). By contrast, core components of the RGF signaling pathway, including *TYROSYLPROTEIN SULFOTRANSFERASE* (*TPST*), *RGF2*, *RGF1 RECEPTOR 2* (*RGFR2*), and *PLT2*, were significantly downregulated at 31°C at 2 DAT, but not at 1 DAT (Fig. 5e). Notably, *RGF1* transcript levels were not significantly altered under nonlethal thermal stress and were therefore not included among the DEGs (Fig. 5; Dataset S2). The *RGF1/2/3* and *RGFR1/2/3* genes are known to function redundantly in root meristem development (Matsuzaki *et al*., 2010; Shinohara *et al*., 2016).

Further analysis showed that *RGF3* and *RGFR3* expression began to decrease at 1 DAT and declined further at 2 DAT (Fig. 5f). Together, these results indicate that nonlethal thermal stress suppresses key components of the RGF‐RGFR‐PLT2 signaling pathway at the stage when meristem reduction becomes apparent. These RNA‐seq results were independently validated by qPCR (Fig. S2).

These findings suggest that roots adapt to nonlethal thermal stress primarily through modulation of developmental gene programs rather than activation of canonical stress‐response pathways. Zone‐resolved transcriptome analysis revealed a coordinated transcriptional shift in which genes associated with meristem maintenance and cell proliferation were downregulated, while genes associated with differentiation were concomitantly upregulated (Figs 5, S2). In particular, genes involved in endodermal maturation, cell wall modification, and Casparian strip formation were selectively induced at 2 DAT, indicating that nonlethal thermal stress actively promotes differentiation rather than simply suppressing growth (Fig. 5b,d).

To further test whether this response differs from canonical heat‐shock signaling, we examined representative heat‐shock marker genes, including *HSFA2*, *HSFA7a*, *HSP70‐1*, and *HSP101* (Schramm *et al*., 2006; Charng *et al*., 2007; Liu *et al*., 2011; Yoshida *et al*., 2011). These genes are well‐established downstream targets of *HSFA1* transcription factors and are strongly induced during acute heat stress. Direct comparison with a published heat‐shock dataset at 22 and 44°C for 3 h (Yang *et al*., 2023) confirmed that these genes were highly induced under heat shock but exhibited little or no induction under nonlethal thermal stress (Fig. S3).

Together, these results demonstrate that nonlethal thermal stress triggers a regulatory program distinct from acute heat shock, characterized by developmental reprogramming rather than activation of the canonical *HSFA1*‐*HSP* pathway. This zone‐specific transcriptional shift provides a mechanistic framework linking environmental temperature to the modulation of developmental signaling pathways, such as the RGF‐RGFR‐PLT2 module, thereby contributing to root adaptation under nonlethal thermal stress.

### The *rgf1/2/3*, *rgfr1/2/3*, and *plt2* mutants are hypersensitive to nonlethal thermal stress

Together with the phenotypic data shown in Figs 1, 2, 3, 4, our transcriptomic findings (Fig. 5) suggested that the RGF peptide‐RGF receptor‐PLT2 signaling pathway controls root meristem size during adaptation to nonlethal thermal stress. To test this idea, we examined meristem size and O_2_˙^−^ accumulation in *rgf1/2/3*, *rgfr1/2/3*, and *plt2* mutants exposed to nonlethal thermal stress.

**Fig. 6:**
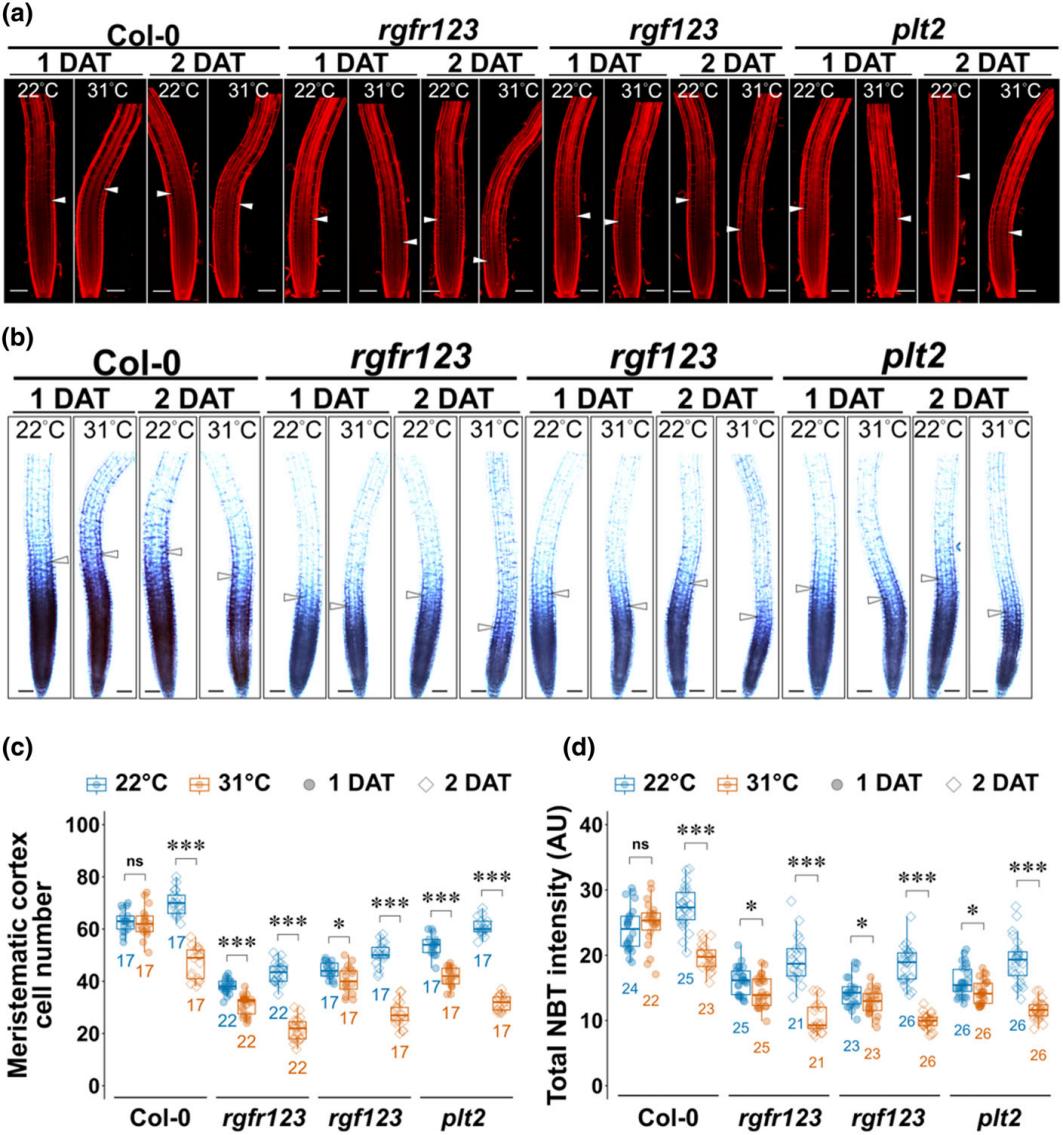
The *rgf1/2/3*, *rgfr1/2/3*, and *plt2* mutants of *Arabidopsis thaliana* are hypersensitive to nonlethal thermal stress. (a) Confocal images of roots from wild‐type (Col‐0) and mutant lines at 1 or 2 d DAT to control (22°C) or heat‐stress (31°C) conditions. Roots were stained with propidium iodide before imaging. (b) Light microscope images of roots from designated genotypes at 1 or 2 DAT to control (22°C) or heat‐stress (31°C) conditions, followed by nitroblue tetrazolium staining. NBT signals were evaluated within the anatomically defined meristematic zone. The arrowheads in (a) and (b) indicate the junction between the meristematic and elongation zones. Bars, 100 μm. (c, d) Quantitative analysis of (a) and (b), respectively. For NBT staining, total signal intensity within the meristematic zone was quantified. Asterisks indicate the heat treatment group is significantly different from the control (*t*‐test or Wilcoxon rank sum test, depending on the data normality; ***, *P* < 0.001; *, *P* < 0.05), while ns indicates nonsignificant differences (*P* > 0.05). In the boxplots, the circles represent each individual data point, and sample sizes are shown below the corresponding boxes. The horizontal line within the boxes represents the median, and the upper and lower hinges, respectively, indicate the 75^th^ and 25^th^ percentiles (interquartile range, IQR). The whiskers extend to 1.5 × the IQR.

In wild‐type seedlings, the meristematic zone size at 31°C was comparable to that at 22°C at 1 DAT, but was significantly reduced by 2 DAT (Fig. 6a,c). By contrast, in both *rgf1/2/3* and *rgfr1/2/3* mutants, the relative meristematic zone size at 31°C was already reduced at 1 DAT and decreased further by 2 DAT (Fig. 6a,c). A similar trend was observed for O_2_˙^−^ levels: in the wild‐type, NBT staining at 31°C was indistinguishable from that at 22°C at 1 DAT, but decreased by 2 DAT (Fig. 6b,d). However, in the *rgf1/2/3* and *rgfr1/2/3* mutants, O_2_˙^−^ signals under nonlethal thermal stress compared with the control temperature showed weaker O_2_˙^−^ signals that were already reduced at 1 DAT and became strongly diminished by 2 DAT (Fig. 6b,d). Similarly, in the *plt2* mutant, the relative meristematic zone size and O_2_˙^−^ accumulation under nonlethal thermal stress were reduced at both 1 and 2 DAT compared with the wild‐type (Fig. 6a–d). Together, these findings demonstrated that the *rgf1/2/3*, *rgfr1/2/3*, and *plt2* mutants showed hypersensitive phenotypes in meristematic zone size and O_2_˙^−^ levels under nonlethal thermal stress compared with the control temperature, consistent with a role for the RGF‐RGFR‐PLT2 signaling pathway in maintaining meristem size and redox balance under elevated temperature.

### RGF1 peptide treatment mitigates nonlethal thermal stress and restores root meristem size

The downregulation of the RGF‐RGFR‐PLT2 pathway genes in the wild‐type (Fig. 5e,f) coincided with reduced meristem size and altered O_2_˙^−^ levels in root developmental zones at 2 DAT under nonlethal thermal stress. Notably, in *rgf1/2/3*, *rgfr1/2/3*, and *plt2* mutants, similar phenotypes were already evident at 1 DAT, indicating enhanced sensitivity to thermal stress (Fig. 6). Together, these observations suggest that nonlethal thermal stress impairs root meristem development by inhibiting this pathway and thereby altering redox states. Given that ROS act downstream of the RGF‐RGFR‐PLT2 signaling cascade, these results further suggest that the observed redox changes are a consequence of pathway suppression.

We therefore hypothesized that exogenous RGF1 application could counteract these defects by activating the RGF‐RGFR‐PLT2 pathway at 2 DAT, when thermal stress‐induced defects become apparent. Although RGF1 transcript levels were not significantly altered under nonlethal heat stress, we used RGF1 for the rescue experiments because RGF1, RGF2, and RGF3 function redundantly within the same signaling pathway regulating root meristem maintenance. RGF1 was selected because its role in ROS‐mediated meristem regulation has been mechanistically characterized in previous studies (Yamada *et al*., 2020), whereas the ROS‐related functions of RGF2 and RGF3 remain unclear. RGF1 has been widely used as a representative ligand to probe pathway activity *in vivo*, allowing us to test whether reactivation of the RGF‐RGFR‐PLT2 pathway can counteract heat‐induced developmental defects.

**Fig. 7:**
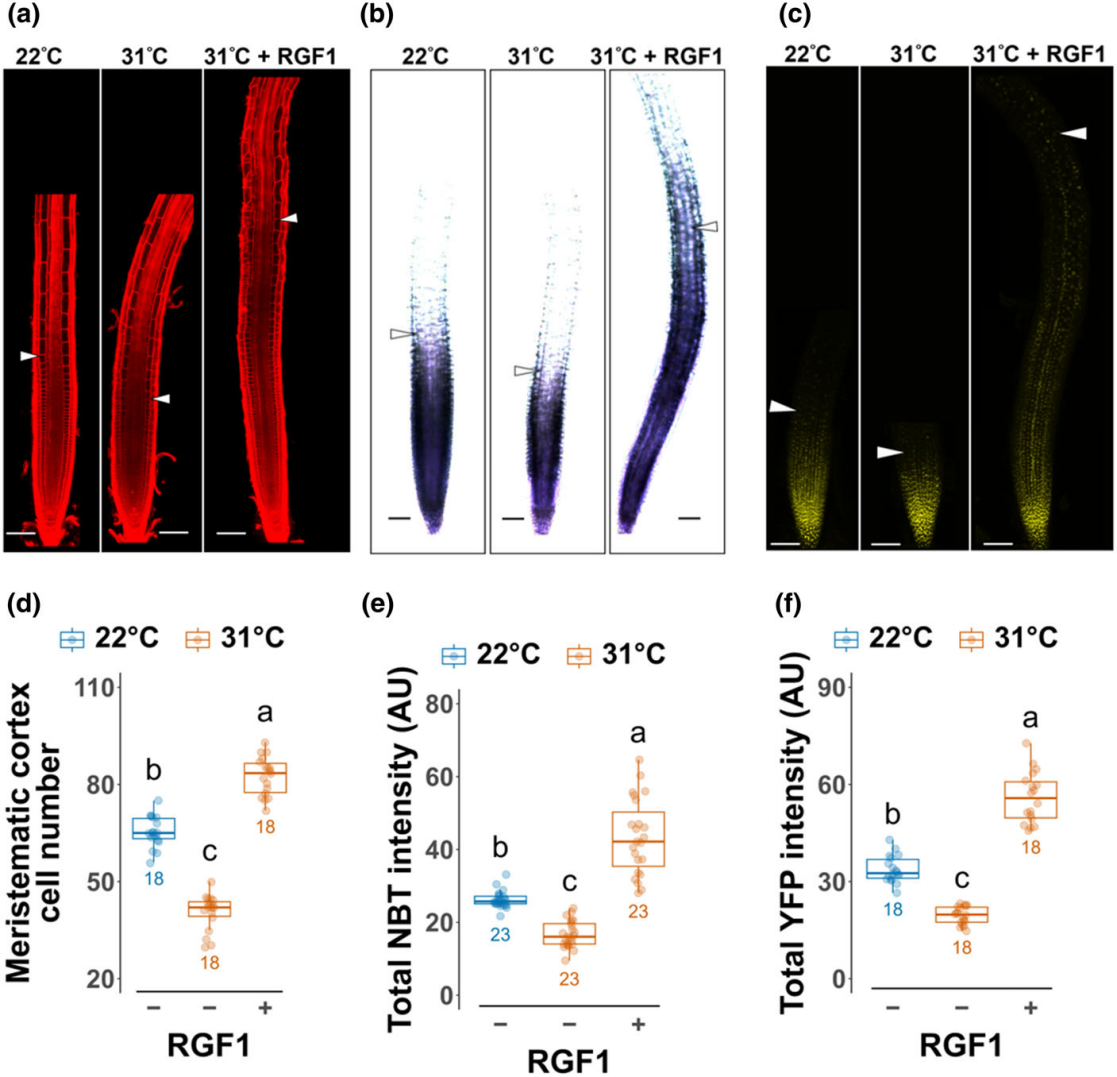
RGF1 treatment initiated at 1 DAT restores meristem function under nonlethal thermal stress in *Arabidopsis thaliana*. (a) Confocal images of gPLT2‐YFP roots 2 DAT to control (22°C) or heat‐stress (31°C) conditions with or without 5 nM RGF1 treatment; roots were stained with propidium iodide. (b) Light microscope images of *gPLT2‐YFP* roots 2 DAT to 22 or 31°C conditions with or without 5 nM RGF1 treatment; stained with nitroblue tetrazolium. Arrowheads in (a, b) mark the junction between the meristematic and elongation zones. (c) Confocal images of *gPLT2‐YFP* roots 2 DAT to the indicated conditions. The confocal images in (a) and (c) are composed of multiple stitched tiles, set against a black background. Bars, 100 μm. (d–f) Quantitative analysis of images in (a–c). Different letters represent significant differences using Fisher's ANOVA + REGWQ test (d), ART‐ANOVA + ART‐Contrast test (e), or Welch's ANOVA + Games–Howell test (f), *P* < 0.05. In the boxplots, the circles represent each individual data point, and sample sizes are shown below the corresponding boxes. The horizontal line within boxes represents the median, and the upper and lower hinges, respectively, indicate the 75^th^ and 25^th^ percentiles (interquartile range, IQR). The whiskers extend to 1.5 × the IQR.

To test this hypothesis, we grew the seedlings of the *gPLT2‐YFP* reporter line (Galinha *et al*., 2007) for 7 d at 22°C, transferred them to 22 or 31°C, and applied mock or 5 nM RGF1 peptide treatment at 2 DAT at 31°C. At 3 DAT, we assessed meristem size and O_2_˙^−^ accumulation. To monitor whether RGF1 restores the RGF‐RGFR‐PLT2 pathway, we also assessed PLT2‐YFP protein localization. In mock‐treated seedlings at 31°C, meristem size was reduced compared with controls at 22°C (Fig. 7a,d). However, RGF1‐treated seedlings showed a significantly larger meristem, even under heat stress (Fig. 7a,d). Consistently, O_2_˙^−^ accumulation was higher with RGF1 treatment than with mock treatment at 31°C (Fig. 7b,e). Nonlethal thermal stress also restricted the PLT2 protein localization domain compared with control conditions (Fig. 7c,f). Strikingly, RGF1 application expanded PLT2 distribution despite the stress (Fig. 7c,f). Together, these results demonstrate that RGF1 treatment mitigates the effects of nonlethal heat stress on O_2_˙^−^ production, meristem size, and PLT2 protein accumulation and distribution.

To further examine redox dynamics, we performed rescue experiments using the GRX‐roGFP2 redox biosensor (Meyer *et al*., 2007). Consistent with our previous observations (Fig. 4), nonlethal thermal stress induced premature oxidation and decreased the extent of the reduced domain in the root tip (Fig. S4a,b). In mock‐treated seedlings, cells with a higher RO index appeared closer to the root tip at 31°C compared with 22°C (Fig. S4a). By contrast, RGF1 treatment delayed the appearance of cells with a higher RO index even under nonlethal thermal stress (Fig. S4b). Accordingly, the distance from the QC to the first detectable cells with a higher RO index was increased by RGF1 treatment (Fig. S4a–c).

Furthermore, we examined the mean RO index in the meristematic zone located proximal to the first detectable cells with a higher RO index under nonlethal thermal stress following RGF1 treatment (Fig. S4d). Notably, RGF1 restored a lower RO index and a more reduced state in this region even under nonlethal thermal stress (Fig. S4d). Together, these results indicate that RGF1 application counteracts stress‐induced redox imbalance, likely by restoring downstream ROS dynamics of the RGF‐RGFR‐PLT2 pathway, thereby stabilizing root meristem function under elevated temperature.

### The *rgf1/2/3*, *rgfr1/2/3*, and *plt2* mutants exhibit hypersensitive lateral root growth defects under nonlethal thermal stress

**Fig. 8:**
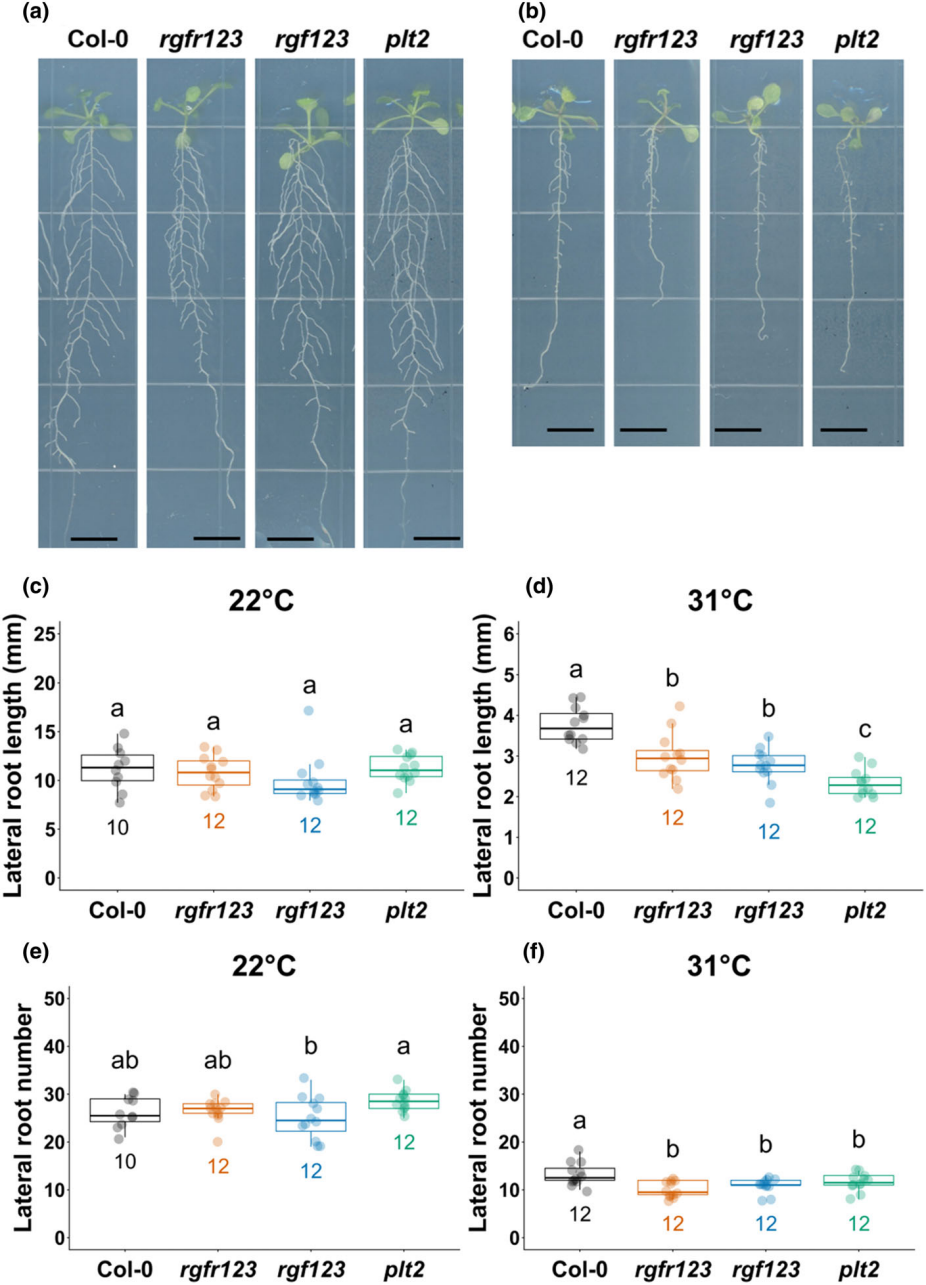
The lateral roots of *rgf1/2/3*, *rgfr1/2/3*, and *plt2* mutants of *Arabidopsis thaliana* are hypersensitive to nonlethal thermal stress. (a, b) Images of wild‐type (Col‐0) and mutant lines were taken after transfer to (a) 22°C or (b) 31°C for 6 d. Bars, 10 mm. Quantification of average lateral root length (c, d) and number (e, f) per seedling under the indicated conditions. Different letters represent significant differences using ART‐ANOVA + Tukey–Kramer test (c), Fisher's ANOVA + REGWQ test (d), Fisher's ANOVA + Tukey–Kramer test (e), and Fisher's ANOVA + REGWQ test (f), *P* < 0.05. In the boxplots, the circles represent each individual data point, and sample sizes are shown below the corresponding boxes. The horizontal line within boxes represents the median, and the upper and lower hinges, respectively, indicate the 75^th^ and 25^th^ percentiles (interquartile range, IQR). The whiskers extend to 1.5 × the IQR.

We investigated nonlethal thermal stress adaptation in the primary root meristem at the early seedling stage (7‐ to 9‐d‐old) (Figs 1, 2, 3, 4, 6, 7). Because nonlethal thermal stress impaired primary root meristem function at the early seedling stage, we hypothesized that these effects might persist into later developmental stages (13‐d‐old seedlings), potentially affecting both primary root elongation and lateral root development. We next investigated thermal stress adaptation in lateral roots. Because the *rgf1/2/3*, *rgfr1/2/3*, and *plt2* mutants exhibited hypersensitive responses to nonlethal thermal stress, showing smaller root meristems and reduced superoxide accumulation (Fig. 6), we wondered whether they would show a similar hypersensitive response in lateral root development. To address this question, we examined their lateral root phenotypes at 6 DAT (13‐d‐old seedlings) under both control (22°C) and nonlethal thermal stress (31°C) conditions (Fig. 8).

At 22°C, 13‐d‐old *rgf1/2/3*, *rgfr1/2/3*, and *plt2* mutant seedlings developed lateral roots comparable to those of the wild‐type (Fig. 8a,c). By contrast, exposure to 31°C markedly restricted lateral root elongation in the mutants, resulting in simpler root systems with markedly shorter lateral roots at 6 DAT compared with those grown at 22°C (Fig. 8b,d). By contrast, lateral root number was less strongly affected under nonlethal thermal stress (Fig. 8e,f).

Thus, the r*gf1/2/3*, *rgfr1/2/3*, and *plt2* mutants exhibited more pronounced defects than the wild‐type in both primary root growth and lateral root elongation under nonlethal thermal stress.

### The RGF2 and RGF8 peptide treatments stimulate lateral root growth under extended nonlethal thermal stress

**Fig. 9:**
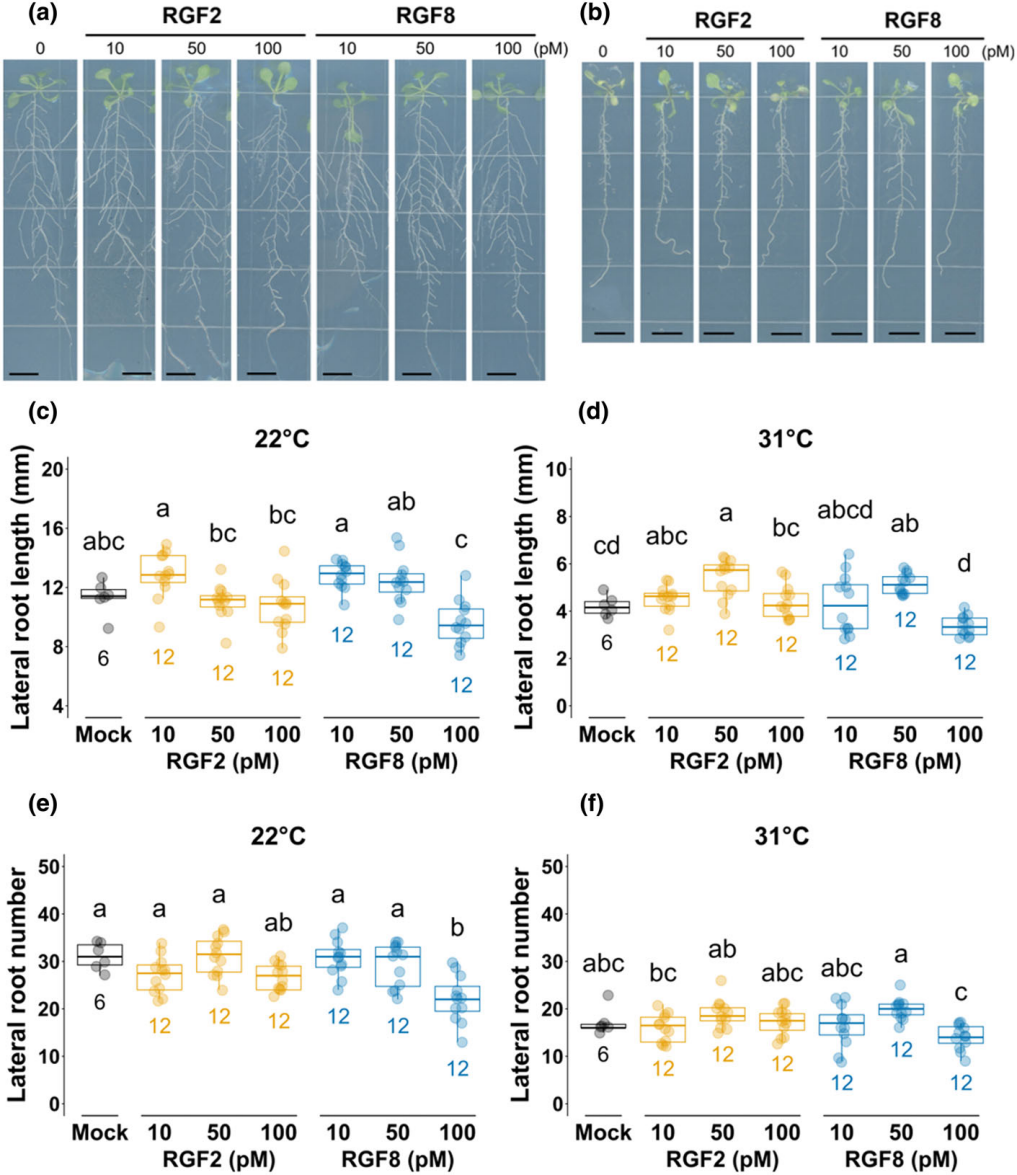
RGF2 and RGF8 peptide treatments stimulate lateral root growth under nonlethal thermal stress in *Arabidopsis thaliana*. (a, b) Light microscope images taken after wild‐type seedlings were transferred to the indicated concentrations of RGF2 or RGF8 peptides for 6 d under (a) 22°C or (b) 31°C conditions. Bars, 10 mm. Quantification of average lateral root length (c, d) and number (e, f) per seedling under the indicated conditions. Lateral root length and number were measured in seedlings treated as shown in (a) and (b), respectively. (c–f) Quantitative analysis of (a, b). Different letters represent significant differences using Fisher's ANOVA + Tukey–Kramer test (c, e, f), and Welch's ANOVA + Games–Howell test (d), *P* < 0.05. In the boxplots, the circles represent each individual data point, and sample sizes are shown below the corresponding boxes. The horizontal line within boxes represents the median, and the upper and lower hinges, respectively, indicate the 75^th^ and 25^th^ percentiles (interquartile range, IQR). The whiskers extend to 1.5 × the IQR.

We hypothesized that RGF2 and RGF8 mitigate lateral root growth inhibition during extended nonlethal thermal stress because these two peptide genes were notably downregulated under nonlethal thermal stress in our transcriptome analysis (Fig. 5e). To test this hypothesis, we examined whether the RGF2 and RGF8 peptides can also alleviate lateral root development inhibition during extended nonlethal thermal stress, beyond alleviating primary root meristem inhibition. Seedlings were grown for 7 d at 22°C and then transferred to either the control temperature (22°C) or the nonlethal thermal stress condition (31°C). Plants were subsequently mock‐treated or exposed to RGF2 or RGF8 for 6 d (6 DAT; 13‐d‐old seedlings) (Fig. 9). Roots produced numerous and elongated lateral roots under the control condition (22°C), and treatment with low concentrations (10, 50, and 100 pM) of the RGF2 or RGF8 peptides had little effect on lateral root growth (Fig. 9a,c). Exposure to 31°C with mock treatment markedly suppressed lateral root growth (Fig. 9a–d). However, treatment with 50 pM RGF2 or RGF8 substantially restored lateral root development under heat stress, alleviating the growth inhibition observed at 31°C (Fig. 9b,d). Interestingly, these low peptide concentrations had only minor effects under control conditions (Fig. 9a,c), but strongly promoted lateral root elongation under nonlethal thermal stress (Fig. 9b,d). Taken together, these data suggest that the defects in lateral root development under nonlethal thermal stress are at least partly attributable to the downregulation of RGF2 and RGF8, and that RGF2 and RGF8 treatments enhance lateral root growth under nonlethal thermal stress. Thus, RGF peptides not only sustain primary root meristem activity but also promote lateral root development, resulting in a more elaborate and potentially more resilient root system under nonlethal thermal stress. By contrast, RGF2 and RGF8 peptide treatments had relatively minor effects on lateral root number (Fig. 9e,f).

## Discussion

A deeper understanding of how nonlethal thermal stress reshapes root developmental programs will be essential for developing strategies to enhance crop resilience and maintain productivity under future climate scenarios.

### Defining 31°C as a nonlethal thermal stress condition

Defining nonlethal thermal stress conditions is an important step in better understanding how plants can be expected to respond and adapt to gradual temperature increases associated with climate change. The observed decline in meristem activity and overall root system architecture at elevated but nonlethal temperatures highlights the need to define precise thresholds for nonlethal stress in plant development. From an agricultural viewpoint, such thresholds may represent hidden constraints that limit crop performance as temperatures rise. Importantly, root growth inhibition occurred in a graded manner across the tested temperature range rather than at a discrete threshold. Root growth was only mildly affected at 30°C, substantially reduced at 31°C, and nearly arrested at 32°C (Fig. 1). Temperatures at which root growth is completely arrested are unlikely to represent an adaptive developmental response and may instead reflect a failure to sustain meristem function, resembling severe or near‐lethal heat stress conditions examined in previous studies. Based on this graded response, we selected 31°C as a representative nonlethal, growth‐restrictive condition under which roots remain viable and continue to grow, albeit at a reduced rate. This temperature therefore provides an experimental window to examine active developmental and physiological adaptation rather than irreversible damage.

In this study, we identified 31°C as a nonlethal thermal stress condition for Arabidopsis root development. Unlike lethal heat shock (above 37°C), which rapidly causes seedling death, exposure to 31°C allowed plants to survive but resulted in distinct developmental impairments. Nonlethal thermal stress reduced primary root growth, suppressed O_2_˙^−^ accumulation, shifted the redox balance from a reduced to a more oxidized state, decreased root meristem size, and restricted PLT2 protein distribution at the seedling stage (Figs 1, 2, 3, 4, 7). Although the overall structure of the apical meristem remained intact, cell division and progression within the meristematic zone markedly slowed down, leading to an earlier transition toward cell elongation and differentiation (Figs 3, S1). A transcriptome analysis of root developmental zones further showed the downregulation of the genes related to cell proliferation and meristem maintenance, together with upregulation of differentiation‐related genes, rather than induction of canonical heat‐shock response genes (Figs 5, S2, S3).

Seedlings could adapt and survive under nonlethal thermal stress but eventually developed shorter primary roots with shorter lateral roots (Figs 1, 8). Thus, while nonlethal thermal stress does not cause immediate death, its adaptive cost becomes evident as diminished growth and productivity (Figs 1, 8).

### Nonlethal thermal stress modulates redox state and root development

Using the GRX‐roGFP2 redox biosensor, we demonstrate that nonlethal thermal stress induces spatially distinct changes in cellular redox states along the root developmental axis (Fig. 4). Our earlier analyses (Figs 1, 2, 3) revealed a strong association between changes in O_2_˙^−^ distribution and alterations in root developmental zones under nonlethal thermal stress, leading us to hypothesize that intracellular redox states are differentially regulated across distinct developmental zones. In our previous studies, O_2_˙^−^ and H_2_O_2_ were analyzed separately using distinct detection methods (Yamada *et al*., 2020); however, it remained unclear to what extent these ROS changes are reflected in the overall intracellular redox state. Although analysis of ROS localization is important, it has remained unclear whether these ROS changes are sufficient to alter intracellular redox states *in vivo*. Although GRX‐roGFP2 does not directly measure individual ROS species, it enabled visualization of intracellular redox status in living tissues under conditions where conventional chemical staining methods were technically limited due to accelerated differentiation, which reduced probe penetration. Accelerated differentiation likely promoted the formation of apoplastic barriers such as the Casparian strip, thereby preventing reliable detection of H_2_O_2_. Consistent with this interpretation, multiple genes associated with Casparian strip formation were transcriptionally induced under nonlethal thermal stress (Fig. 5d).

Using this system, we found that the position of oxidized cells shifted closer to the root tip under 31°C, indicating that redox regulation is dynamically reorganized across developmental zones in response to elevated temperature.

Furthermore, exogenous application of RGF1 shifted the appearance of oxidized cells toward more distal regions from the root tip under both 22 and 31°C conditions, indicating suppression of oxidation in the proximal meristematic region (Fig. S4). This RGF1‐induced redistribution of redox states suggests that peptide signaling can re‐establish redox balance along the developmental axis, thereby counteracting the meristem size reduction and altered root developmental patterns caused by nonlethal thermal stress.

This zone‐specific redistribution of redox states was closely associated with reduced meristem size and altered root developmental patterns, suggesting that redox signaling functions as a key integrator linking environmental temperature cues to developmental regulation. Importantly, these results indicate that ROS signals are not simply reflected as uniform changes in cellular redox state, but are integrated into spatially patterned redox dynamics along the developmental axis. Notably, these changes occurred under nonlethal thermal conditions, where canonical heat stress responses were not strongly activated, highlighting a distinct mode of temperature adaptation.

Together, our findings reveal that redox dynamics are spatially patterned along the root developmental axis, providing a mechanistic framework for how roots coordinate growth and stress adaptation under nonlethal thermal stress.

### Developmental zone‐specific transcriptomics reveals root adaptation to nonlethal thermal stress

Most previous transcriptomic studies on heat stress in Arabidopsis have focused on the induction of classical stress‐responsive genes under acute or moderately high temperatures (Larkindale & Vierling, 2008; Quint *et al*., 2016; Vu *et al*., 2019; Guo *et al*., 2022). Consequently, most RNA‐seq analyses have been performed on whole seedlings or entire roots, providing valuable global insights but lacking spatial resolution within distinct developmental zones. However, our approach targeted specific root zones at defined time points before and after the onset of accelerated developmental transition associated with slowed root growth, allowing us to capture the earliest stages of adaptation to nonlethal heat stress. This strategy is particularly important because transcriptional responses in the root apex, especially in the meristematic zone, are often masked in whole‐root datasets dominated by mature tissues (Li *et al*., 2016).

This developmental zone‐ and time‐resolved perspective reveals that roots respond to 31°C primarily through developmental signaling pathways (Figs 5, S2), rather than by strongly activating canonical heat stress response genes (Fig. S3). Importantly, our analysis revealed clear zone‐specific differences in transcriptional responses that were not apparent in whole‐root datasets. In particular, genes associated with the RGF‐RGFR‐PLT2 pathway were preferentially downregulated in the meristematic zone, whereas genes upregulated at 31°C were enriched in differentiation‐related processes, including phenylpropanoid metabolism, Casparian strip formation, and ethylene‐responsive pathways (Figs 5, S2). Notably, this approach enabled us to simultaneously capture both transcriptional repression of meristem‐maintenance programs and activation of differentiation‐associated genes, providing mechanistic insight into how root developmental programs are reorganized under nonlethal thermal stress. However, how moderately elevated temperature is sensed and transduced into transcriptional repression of the RGF‐RGFR‐PLT2 pathway remains unclear. Understanding this process will be important for elucidating how developmental signaling pathways integrate moderate temperature cues into root growth regulation.

### Nonlethal thermal stress shifts the effective range of RGF signaling

A previous study showed that overexpression of RGF8 or treatment with high concentrations (2–4 μM) inhibits lateral root development under normal growth conditions (Fernandez *et al*., 2015). By contrast, our treatments used much lower concentrations (10–100 pM). These low concentrations had no effect under the control condition, but 50 pM RGF2 and RGF8 markedly restored lateral root elongation under nonlethal thermal stress (Fig. 8). However, 10 pM of these peptides had no effect, whereas 100 pM had negative effects. These observations suggest that RGF peptides possess a temperature‐dependent signaling threshold, with nonlethal thermal stress shifting the effective signaling range to extremely low concentrations *c*. 50 pM. The molecular mechanism underlying this temperature‐dependent modulation of RGF signaling sensitivity remains unknown and will need to be elucidated in future studies.

### Exogenous application of RGF peptides modulated root system architecture, primarily by promoting lateral root growth, under nonlethal thermal stress

Under nonlethal thermal stress, topical application of RGF peptides promoted not only primary root meristem maintenance but also lateral root growth, thereby modulating root system architecture. Consistent with their distinct physiological roles, RGF1 primarily acted on the primary root meristem, whereas RGF8 mainly regulated lateral root development under nonlethal thermal stress (Figs 7, 8). Interestingly, RGF8 differs from RGF1 and RGF2 in that it cannot rescue the *tpst* mutant primary meristem defect (Matsuzaki *et al*., 2010), consistent with its specialized role in lateral root development. Together, these findings suggest that roots adapt to nonlethal thermal stress by differentially modulating the functions of primary‐ and lateral‐root‐associated RGF peptides.

The enhanced lateral root growth suggests that RGF signaling acts beyond the root tip, influencing post‐meristematic developmental programs that shape overall root system architecture. These findings further suggest that coordinated regulation of distinct RGF peptides may contribute to the maintenance of root system architecture under nonlethal thermal stress by balancing primary meristem activity and lateral root growth.

### Potential of RGF peptide application to mitigate heat stress in crops

RGF1 and RGF8 peptides could serve as a potential tool to alleviate thermal stress without the need for genetic modification. Because these small peptides are stable and biologically active at even low concentrations (50 pM) (Fig. 8), topical application may provide a flexible and rapid means to enhance stress resilience in plants. RGF peptide treatment lowered H_2_O_2_ levels (Yamada *et al*., 2020), which are typically elevated under various stress conditions, suggesting that this pathway could alleviate not only heat but also other abiotic stresses involving oxidative imbalance. Although peptide delivery through soil remains technically challenging, our successful application on agar medium indicates that this approach could be adapted to hydroponically cultivated crops such as rice and other water‐grown species.

In our study, exogenous RGF1 treatment expanded the root meristem and conferred resistance to nonlethal thermal stress (31°C) (Fig. 7). By contrast, the roots of the *rgf1/2/3* and *rgfr1/2/3* mutants, which possess smaller meristems, were more susceptible to the thermal stress (Fig. 6). These results demonstrate that root meristem size is a critical determinant of the ability to withstand nonlethal thermal stress.

It is plausible that similar regulatory mechanisms function across diverse crops. *RGF* and *RGFR* genes are conserved in at least 16 species of angiosperms, including *Oryza sativa*, *Zea mays*, *Solanum lycopersicum*, and *Glycine max* (Fang *et al*., 2021). The mature peptide sequences are also highly conserved across species, and synthetic *Zea mays* RGF1 (ZmRGF1) peptide or overexpression of the *ZmRGF1* gene enlarges the meristematic zone in Arabidopsis roots (Fang *et al*., 2021). If the RGF‐RGFR signaling pathway is both evolutionarily and functionally conserved, it may be possible to use Arabidopsis RGF peptide to alleviate abiotic stress in crops.

In this research, we demonstrated that RGF1, RGF2, and RGF8 peptides mitigated defects of primary root meristem and lateral root elongation under nonlethal thermal stress. By contrast, the *rgf1/2/3*, *rgfr1/2/3*, and *plt2* mutants showed hypersensitivity to the stress. Together, our findings highlight the potential of RGF peptide signaling as a natural, evolutionarily conserved mechanism that links developmental plasticity with environmental adaptation. Harnessing this pathway may provide a new foundation for enhancing plant resilience in the era of global climate change.

## Competing interests

None declared.

## Author contributions

Y‐CH and MY conceptualized the study; Y‐CH, S‐YS and MY performed all experiments; J‐KL performed the computational and statistical analyses; all the authors wrote the paper.

References

Charng
YY
, 
Liu
HC
, 
Liu
NY
, 
Chi
WT
, 
Wang
CN
, 
Chang
SH
, 
Wang
TT
. 2007. A heat‐inducible transcription factor, HsfA2, is required for extension of acquired thermotolerance in Arabidopsis. Plant Physiology
143: 251–262.17085506
10.1104/pp.106.091322PMC1761974

Dunand
C
, 
Crevecoeur
M
, 
Penel
C
. 2007. Distribution of superoxide and hydrogen peroxide in Arabidopsis root and their influence on root development: possible interaction with peroxidases. New Phytologist
174: 332–341.17388896
10.1111/j.1469-8137.2007.01995.x

Fang
Y
, 
Chang
J
, 
Shi
T
, 
Luo
W
, 
Ou
Y
, 
Wan
D
, 
Li
J
. 2021. Evolution of RGF/GLV/CLEL peptide hormones and their roles in land plant growth and regulation. International Journal of Molecular Sciences
22: 13372.34948169
10.3390/ijms222413372PMC8708909

Fernandez
A
, 
Drozdzecki
A
, 
Hoogewijs
K
, 
Nguyen
A
, 
Beeckman
T
, 
Madder
A
, 
Hilson
P
. 2013. Transcriptional and functional classification of the GOLVEN/ROOT GROWTH FACTOR/CLE‐like signaling peptides reveals their role in lateral root and hair formation. Plant Physiology
161: 954–970.23370719
10.1104/pp.112.206029PMC3561032

Fernandez
A
, 
Drozdzecki
A
, 
Hoogewijs
K
, 
Vassileva
V
, 
Madder
A
, 
Beeckman
T
, 
Hilson
P
. 2015. The GLV6/RGF8/CLEL2 peptide regulates early pericycle divisions during lateral root initiation. Journal of Experimental Botany
66: 5245–5256.26163695
10.1093/jxb/erv329PMC4526922

Fernandez
AI
, 
Vangheluwe
N
, 
Xu
K
, 
Jourquin
J
, 
Claus
LAN
, 
Morales‐Herrera
S
, 
Parizot
B
, 
De Gernier
H
, 
Yu
Q
, 
Drozdzecki
A

*et al*. 2020. GOLVEN peptide signalling through RGI receptors and MPK6 restricts asymmetric cell division during lateral root initiation. Nature Plants
6: 533–543.32393883
10.1038/s41477-020-0645-z

Galinha
C
, 
Hofhuis
H
, 
Luijten
M
, 
Willemsen
V
, 
Blilou
I
, 
Heidstra
R
, 
Scheres
B
. 2007. PLETHORA proteins as dose‐dependent master regulators of Arabidopsis root development. Nature
449: 1053–1057.17960244
10.1038/nature06206

Guo
M
, 
Liu
J
, 
Ma
X
, 
Luo
D
, 
Gong
Z
, 
Lu
M
. 2022. Transcriptome profiling revealed heat stress–responsive genes in *Arabidopsis thaliana*
. Plant Signaling & Behavior
17: 85–95.

Hsiao
YC
, 
Shiue
SY
, 
Yen
MR
, 
Lai
JK
, 
Yamada
M
. 2025. The peptide hormone RGF1 modulates PLETHORA2 stability via reactive oxygen species‐dependent regulation of a cysteine residue. Plant Physiology
198: kiaf244.40608788
10.1093/plphys/kiaf244PMC12225670

Hsiao
YC
, 
Yamada
M
. 2020. The roles of peptide hormones and their receptors during plant root development. Genes
12: 22.33375648
10.3390/genes12010022PMC7823343

Hsiao
YC
, 
Yamada
M
. 2024. Peptide hormones in root growth and development. In: 

Beeckman
TE

, 

Amram
E

, eds. Plant Roots: the Hidden half. Boca Raton, FL, USA: CRC Press.

Jean‐Baptiste
K
, 
McFaline‐Figueroa
JL
, 
Alexandre
CM
, 
Dorrity
MW
, 
Saunders
L
, 
Bubb
KL
, 
Trapnell
C
, 
Fields
S
, 
Queitsch
C
, 
Cuperus
JT
. 2019. Dynamics of gene expression in single root cells of *Arabidopsis thaliana*
. Plant Cell
31: 993–1011.30923229
10.1105/tpc.18.00785PMC8516002

Larkindale
J
, 
Vierling
E
. 2008. Core genome responses involved in acclimation to high temperature. Plant Physiology
146: 748–761.18055584
10.1104/pp.107.112060PMC2245833

Lesk
C
, 
Rowhani
P
, 
Ramankutty
N
. 2016. Influence of extreme weather disasters on global crop production. Nature
529: 84–87.26738594
10.1038/nature16467

Li
S
, 
Yamada
M
, 
Han
X
, 
Ohler
U
, 
Benfey
PN
. 2016. High‐resolution expression map of the Arabidopsis root reveals alternative splicing and lincRNA regulation. Developmental Cell
39: 508–522.27840108
10.1016/j.devcel.2016.10.012PMC5125536

Liao
Y
, 
Smyth
GK
, 
Shi
W
. 2013. The Subread aligner: fast, accurate and scalable read mapping by seed‐and‐vote. Nucleic Acids Research
41: e108.23558742
10.1093/nar/gkt214PMC3664803

Liao
Y
, 
Smyth
GK
, 
Shi
W
. 2014. 
featureCounts: an efficient general purpose program for assigning sequence reads to genomic features. Bioinformatics
30: 923–930.24227677
10.1093/bioinformatics/btt656

Liu
HC
, 
Liao
HT
, 
Charng
YY
. 2011. The role of class A1 heat shock factors (HSFA1s) in response to heat and other stresses in Arabidopsis. Plant, Cell & Environment
34: 738–751.10.1111/j.1365-3040.2011.02278.x21241330

Lobell
DB
, 
Schlenker
W
, 
Costa‐Roberts
J
. 2011. Climate trends and global crop production since 1980. Science
333: 616–620.21551030
10.1126/science.1204531

Love
MI
, 
Huber
W
, 
Anders
S
. 2014. Moderated estimation of fold change and dispersion for RNA‐seq data with DESeq2. Genome Biology
15: 550.25516281
10.1186/s13059-014-0550-8PMC4302049

Lu
L
, 
Liu
H
, 
Wu
Y
, 
Yan
GJ
. 2022. Wheat genotypes tolerant to heat at seedling stage tend to be also tolerant at adult stage: the possibility of early selection for heat tolerance breeding. Crop Journal
10: 1006–1013.

Maeda
H
, 
Fukuyasu
Y
, 
Yoshida
S
, 
Fukuda
M
, 
Saeki
K
, 
Matsuno
H
, 
Yamauchi
Y
, 
Yoshida
K
, 
Hirata
K
, 
Miyamoto
K
. 2004. Fluorescent Probes for Hydrogen Peroxide Based on a Non‐Oxidative Mechanism. Angewandte Chemie International Edition
43: 2389–2391.15114569
10.1002/anie.200452381

Mase
K
, 
Tsukagoshi
H
. 2021. Reactive oxygen species link gene regulatory networks during Arabidopsis root development. Frontiers in Plant Science
12: 660274.33986765
10.3389/fpls.2021.660274PMC8110921

Matsuzaki
Y
, 
Ogawa‐Ohnishi
M
, 
Mori
A
, 
Matsubayashi
Y
. 2010. Secreted peptide signals required for maintenance of root stem cell niche in Arabidopsis. Science
329: 1065–1067.20798316
10.1126/science.1191132

Meng
L
, 
Buchanan
BB
, 
Feldman
LJ
, 
Luan
S
. 2012. CLE‐like (CLEL) peptides control the pattern of root growth and lateral root development in Arabidopsis. Proceedings of the National Academy of Sciences, USA
109: 1760–1765.10.1073/pnas.1119864109PMC327718422307643

Meyer
AJ
, 
Brach
T
, 
Marty
L
, 
Kreye
S
, 
Rouhier
N
, 
Jacquot
JP
, 
Hell
R
. 2007. Redox‐sensitive GFP in *Arabidopsis thaliana* is a quantitative biosensor for the redox potential of the cellular glutathione redox buffer. The Plant Journal
52: 973–986.17892447
10.1111/j.1365-313X.2007.03280.x

Mittler
R
. 2017. ROS are good. Trends in Plant Science
22: 11–19.27666517
10.1016/j.tplants.2016.08.002

Ohama
N
, 
Sato
H
, 
Shinozaki
K
, 
Yamaguchi‐Shinozaki
K
. 2017. Transcriptional regulatory network of plant heat stress response. Trends in Plant Science
22: 53–65.27666516
10.1016/j.tplants.2016.08.015

Ou
Y
, 
Lu
X
, 
Zi
Q
, 
Xun
Q
, 
Zhang
J
, 
Wu
Y
, 
Shi
H
, 
Wei
Z
, 
Zhao
B
, 
Zhang
X

*et al*. 2016. RGF1 INSENSITIVE 1 to 5, a group of LRR receptor‐like kinases, are essential for the perception of root meristem growth factor 1 in *Arabidopsis thaliana*
. Cell Research
26: 686–698.27229312
10.1038/cr.2016.63PMC4897188

Petricka
JJ
, 
Winter
CM
, 
Benfey
PN
. 2012. Control of Arabidopsis root development. Annual Review of Plant Biology
63: 563–590.10.1146/annurev-arplant-042811-105501PMC364666022404466

Quint
M
, 
Delker
C
, 
Franklin
KA
, 
Wigge
PA
, 
Halliday
KJ
, 
van Zanten
M
. 2016. Molecular and genetic control of plant thermomorphogenesis. Nature Plants
2: 15190.27250752
10.1038/nplants.2015.190

Schindelin
J
, 
Arganda‐Carreras
I
, 
Frise
E
, 
Kaynig
V
, 
Longair
M
, 
Pietzsch
T
, 
Preibisch
S
, 
Rueden
C
, 
Saalfeld
S
, 
Schmid
B

*et al*. 2012. Fiji: an open‐source platform for biological‐image analysis. Nature Methods
9: 676–682.22743772
10.1038/nmeth.2019PMC3855844

Schramm
F
, 
Ganguli
A
, 
Kiehlmann
E
, 
Englich
G
, 
Walch
D
, 
von Koskull‐Doring
P
. 2006. The heat stress transcription factor HsfA2 serves as a regulatory amplifier of a subset of genes in the heat stress response in Arabidopsis. Plant Molecular Biology
60: 759–772.16649111
10.1007/s11103-005-5750-x

Shinohara
H
, 
Mori
A
, 
Yasue
N
, 
Sumida
K
, 
Matsubayashi
Y
. 2016. Identification of three LRR‐RKs involved in perception of root meristem growth factor in Arabidopsis. Proceedings of the National Academy of Sciences, USA
113: 3897–3902.10.1073/pnas.1522639113PMC483324927001831

Song
W
, 
Liu
L
, 
Wang
J
, 
Wu
Z
, 
Zhang
H
, 
Tang
J
, 
Lin
G
, 
Wang
Y
, 
Wen
X
, 
Li
W

*et al*. 2016. Signature motif‐guided identification of receptors for peptide hormones essential for root meristem growth. Cell Research
26: 674–685.27229311
10.1038/cr.2016.62PMC4897187

Thevenaz
P
, 
Unser
M
. 2007. User‐friendly semiautomated assembly of accurate image mosaics in microscopy. Microscopy Research and Technique
70: 135–146.17133410
10.1002/jemt.20393

Tsukagoshi
H
, 
Busch
W
, 
Benfey
PN
. 2010. Transcriptional regulation of ROS controls transition from proliferation to differentiation in the root. Cell
143: 606–616.21074051
10.1016/j.cell.2010.10.020

Ubeda‐Tomas
S
, 
Federici
F
, 
Casimiro
I
, 
Beemster
GT
, 
Bhalerao
R
, 
Swarup
R
, 
Doerner
P
, 
Haseloff
J
, 
Bennett
MJ
. 2009. Gibberellin signaling in the endodermis controls Arabidopsis root meristem size. Current Biology
19: 1194–1199.19576770
10.1016/j.cub.2009.06.023

Ubogoeva
EV
, 
Zemlyanskaya
EV
, 
Xu
J
, 
Mironova
V
. 2021. Mechanisms of stress response in the root stem cell niche. Journal of Experimental Botany
72: 6746–6754.34111279
10.1093/jxb/erab274PMC8513250

Vu
LD
, 
Xu
X
, 
Gevaert
K
, 
De Smet
I
. 2019. Developmental Plasticity at High Temperature. Plant Physiology
181: 399–411.31363006
10.1104/pp.19.00652PMC6776856

Whitford
R
, 
Fernandez
A
, 
Tejos
R
, 
Perez
AC
, 
Kleine‐Vehn
J
, 
Vanneste
S
, 
Drozdzecki
A
, 
Leitner
J
, 
Abas
L
, 
Aerts
M

*et al*. 2012. GOLVEN secretory peptides regulate auxin carrier turnover during plant gravitropic responses. Developmental Cell
22: 678–685.22421050
10.1016/j.devcel.2012.02.002

Xu
S
, 
Hu
E
, 
Cai
Y
, 
Xie
Z
, 
Luo
X
, 
Zhan
L
, 
Tang
W
, 
Wang
Q
, 
Liu
B
, 
Wang
R

*et al*. 2024. Using clusterProfiler to characterize multiomics data. Nature Protocols
19: 3292–3320.39019974
10.1038/s41596-024-01020-z

Yamada
M
, 
Han
X
, 
Benfey
PN
. 2020. RGF1 controls root meristem size through ROS signalling. Nature
577: 85–88.31801996
10.1038/s41586-019-1819-6PMC6930331

Yang
F
, 
Sun
Y
, 
Du
X
, 
Chu
Z
, 
Zhong
X
, 
Chen
X
. 2023. Plant‐specific histone deacetylases associate with ARGONAUTE4 to promote heterochromatin stabilization and plant heat tolerance. New Phytologist
238: 252–269.36631970
10.1111/nph.18729

Yeh
CH
, 
Kaplinsky
NJ
, 
Hu
C
, 
Charng
YY
. 2012. Some like it hot, some like it warm: phenotyping to explore thermotolerance diversity. Plant Science
195: 10–23.22920995
10.1016/j.plantsci.2012.06.004PMC3430125

Yoshida
T
, 
Ohama
N
, 
Nakajima
J
, 
Kidokoro
S
, 
Mizoi
J
, 
Nakashima
K
, 
Maruyama
K
, 
Kim
JM
, 
Seki
M
, 
Todaka
D

*et al*. 2011. Arabidopsis HsfA1 transcription factors function as the main positive regulators in heat shock‐responsive gene expression. Molecular Genetics and Genomics
286: 321–332.21931939
10.1007/s00438-011-0647-7

## Disclaimer

The New Phytologist Foundation remains neutral with regard to jurisdictional claims in maps and in any institutional affiliations.

## Supporting information


**Dataset S1** Primer sequences used in this study.

**Dataset S2** Complete list of differentially expressed genes.

**Dataset S3** Source data for all figures.

**Dataset S4** RNA‐seq source data.

**Fig. S1** Nonlethal thermal stress reduced mitotic cell division marker expression and meristematic zone size.
**Fig. S2** qRT‐PCR validation of RNA‐seq‐identified differentially expressed genes under nonlethal thermal stress.
**Fig. S3** Nonlethal heat stress did not induce heat‐shock responsive genes.
**Fig. S4** RGF1 treatment mitigates redox and developmental zone shifts under nonlethal thermal stress.

**Notes S1** Supplemental protocol for reduced–oxidized index analysis.
Please note: Wiley is not responsible for the content or functionality of any Supporting Information supplied by the authors. Any queries (other than missing material) should be directed to the *New Phytologist* Central Office.

## Data Availability

RNA‐seq data generated in this study have been deposited in the Gene Expression Omnibus (GEO) under accession no. GSE311803 and are publicly available at https://www.ncbi.nlm.nih.gov/geo/query/acc.cgi?acc=GSE311803. Source data and additional materials supporting the findings of this study are provided in Datasets S1–, S4, Figs S1–S4, and Notes S1.
