# Supplementary material for: RGF signaling bridges root development and nonlethal thermal stress adaptation
Source: New Phytol. 2026 Jul 5;251(5):2656–71. doi: 10.1111/nph.71392 (PMC13443300; doi:10.1111/nph.71392)
Supplement: Supplementary file 1 [file NPH-251-2656-s003.pdf]

New Phytologist

***New Phytologist* Supporting Information**

Article title: **RGF signaling bridges root development and non-lethal thermal stress adaptation**

Authors: Yu-Chun Hsiao[1,2], Joon-Keat Lai[1,2], Shiau-Yu Shiue[1,2], and Masashi Yamada[1,2,3] *

Article acceptance date: 11 June 2026

The following Supporting Information is available for this article:

Fig. S1 Non-lethal thermal stress reduced mitotic cell division marker expression and the meristemic zone size.

Fig. S2 qRT-PCR validation of RNA-seq-identified differentially expressed genes under non-lethal thermal stress.

Fig. S3 Non-lethal heat stress did not stimulate the expression of heat-shock responsive genes.

Fig. S4 RGF1 treatment mitigates redox and developmental zone shifts under non-lethal thermal stress.

Note S1 Supplemental protocol for reduced–oxidized (RO) index analysis

Dataset S1 Primer sequences used in this study

Dataset S2 Complete list of differentially expressed genes

Dataset S3 Source data for all figures

Dataset S4 RNA-seq source data

Fig. S1 Non-lethal thermal stress reduced mitotic cell division marker expression and the meristemic zone size. (a) Confocal microscopy was used to capture images of the three developmental zones at 2 days after transfer (DAT) to control (22°C) and heat-stress temperature (31°C) conditions. Arrowheads indicate the boundary between the meristematic zone and the elongation zone, defined according to the established criterion based on cortical cell length, as the region between the quiescent center and the last cortical cell that had not doubled in length relative to its predecessor (see Methods). Representative images are shown; n = 3 independent experiments. Each experiment included more than 15 roots. Scale bar = 100 µm. Quantitative analysis of the distance of the CYCB1;1-GFP signal from QC cells (b) and meristematic cortex cell number (c) in roots grown under the indicated conditions. Meristem size was quantified by counting cortical cell number rather than by area measurement. Asterisks indicate significant differences between the heat treatment group and the control (t-test; ***, $p < 0.001$; **, $p < 0.01$; *, $p < 0.05$), while ns indicates non-significant differences ($p > 0.05$). In the boxplots, the circles represent each individual data point, and sample sizes are shown below the corresponding boxes. The horizontal line within the boxes represents the median, and the upper and lower hinges, respectively, indicate the 75th and 25th percentiles (interquartile range, IQR). The whiskers show the 1.5 extension of the IQR.

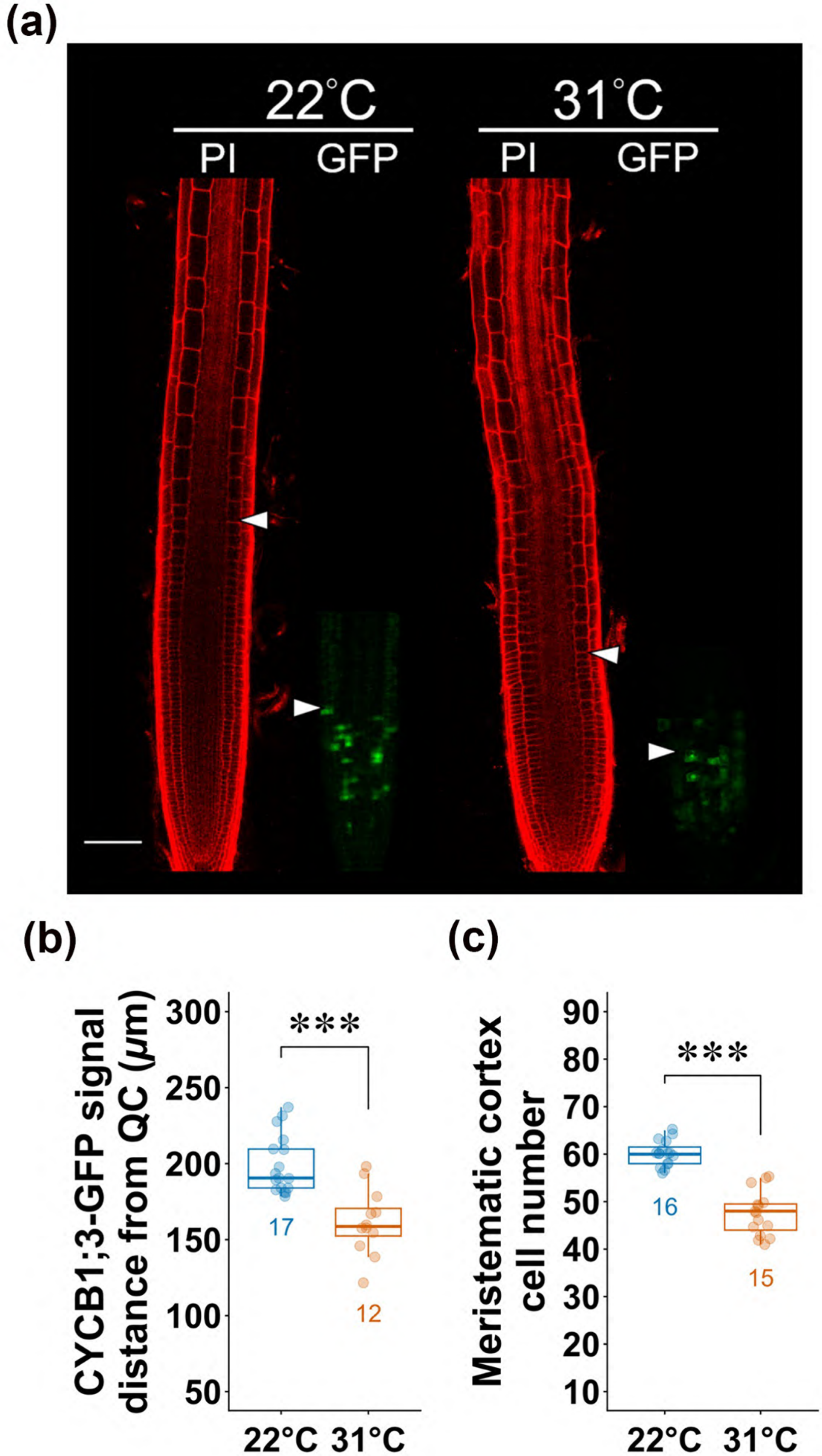

(a)
22°C
31°C
PI
GFP
PI
GFP
(b)
CYCB1;3-GFP signal distance from QC (μm)
300
250
200
150
100
50
***
17
12
22°C
31°C
(c)
Meristematic cortex cell number
90
80
70
60
50
40
30
20
10
***
16
15
22°C
31°C

Fig. S2 qRT-PCR validation of RNA-seq-identified differentially expressed genes under non-lethal thermal stress. (a) RGF1–receptor–PLT2 pathway-related genes from either subset G or O are downregulated exclusively at 2 DAT. (b) Cell elongation- and endodermal differentiation-related genes from subset C, along with the superoxide-detoxifying *SOD1* gene, are up-regulated at 2 DAT. Error bars represent the standard error (SE) ($n=3$; each dot represents 1 biological replicate). The group means were compared using t-test. ***, $p < 0.001$; **, $p < 0.01$; *, $p < 0.05$), while ns indicates non-significant differences ($p > 0.05$).

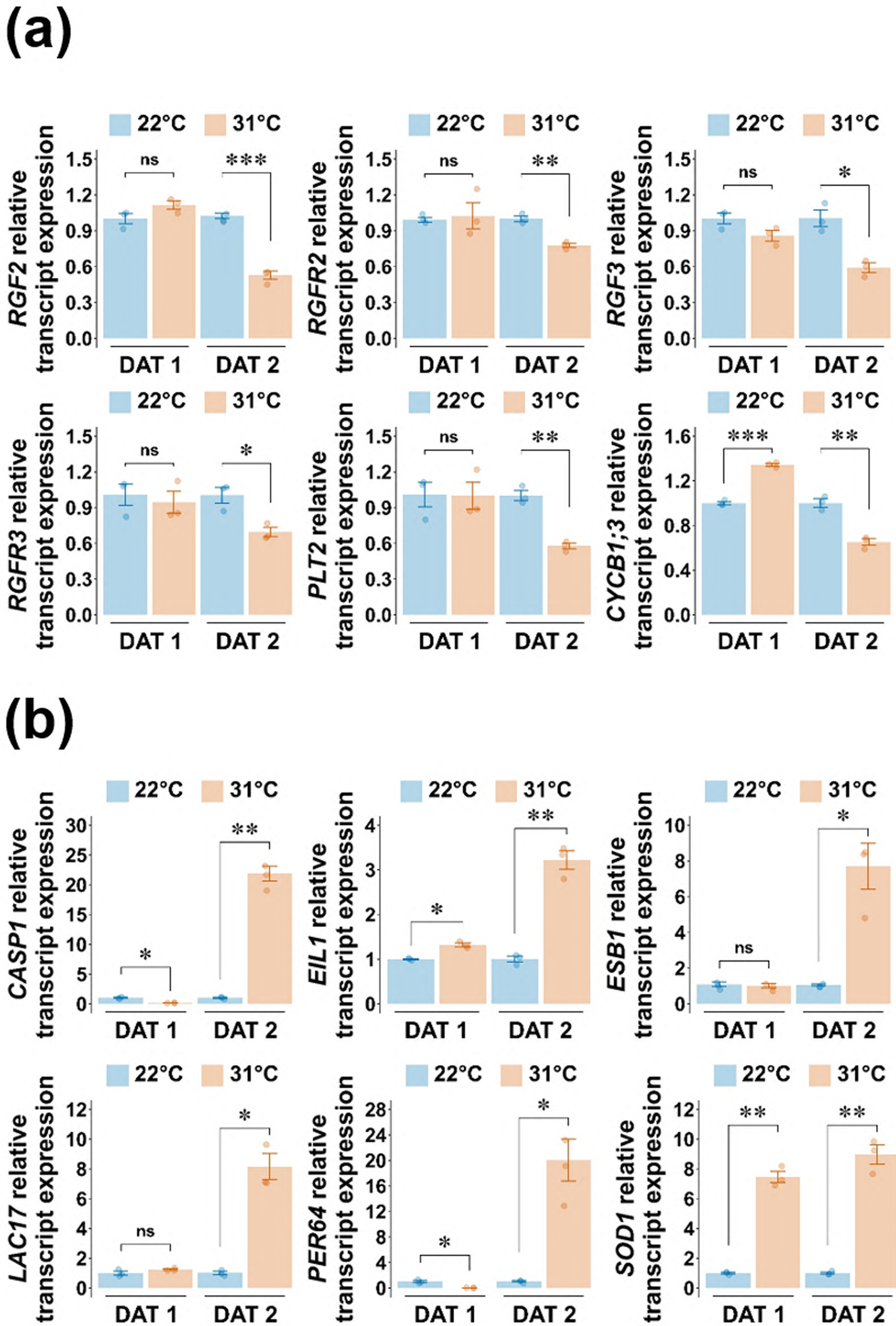
(a)
22°C
31°C
RGF2 relative transcript expression
RGFR2 relative transcript expression
RGF3 relative transcript expression
RGFR3 relative transcript expression
PLT2 relative transcript expression
CYCB1;3 relative transcript expression
ns
***
**
*
DAT 1
DAT 2
(b)
CASP1 relative transcript expression
EIL1 relative transcript expression
ESB1 relative transcript expression
LAC17 relative transcript expression
PER64 relative transcript expression
SOD1 relative transcript expression

Fig. S3 Non-lethal heat stress did not stimulate the expression of heat-shock responsive genes. (a–d) Expression of heat stress marker genes. For the pairwise comparison of the data in this study (boxplots), different letters represent significant differences using ART-ANOVA + Games-Howell test (a), ART-ANOVA + Tukey-HSD test (b), and ANOVA + REGWQ test (c–d). For the two-sample comparison of the data taken from Yang et al., 2023 (bar plots), independent t-test was conducted (a–d). Asterisks indicate the gene expression of the heat treatment group is significantly different from the control (t-test; ***: $p < 0.001$, **: $p < 0.01$, *: $p < 0.05$), while ns indicates non-significant differences ($p > 0.05$). In the boxplots, the circles represent each individual data point, and sample sizes are shown below the corresponding boxes. The horizontal line within boxes represents the median, and the upper and lower hinges, respectively, indicate the 75th and 25th percentiles (interquartile range, IQR). The whiskers show the 1.5 extension of the IQR. In the bagplots, the circles represent each individual data point, and the error bar represent the standard deviation of the samples (n = 3).

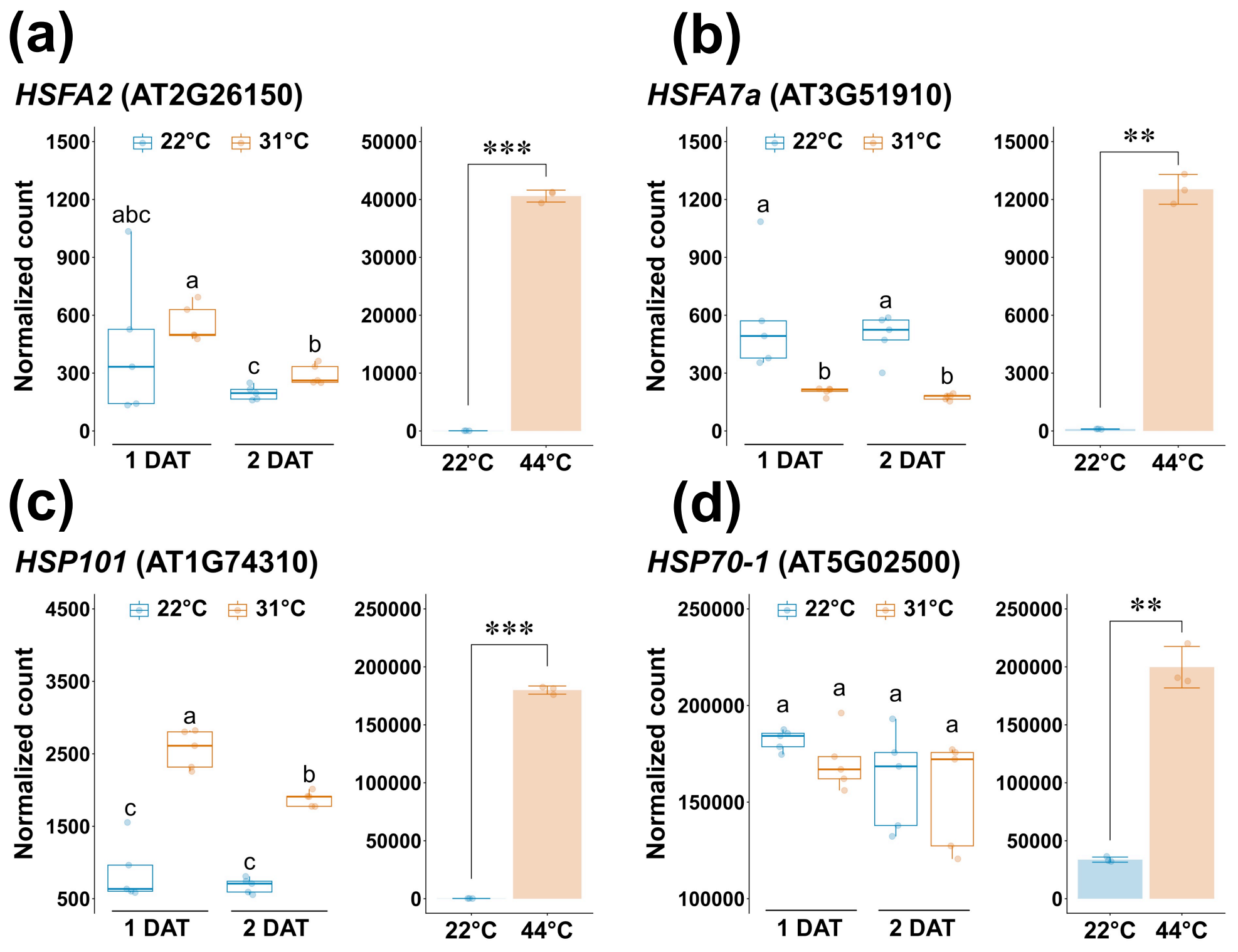

Fig. S4 RGF1 treatment mitigates redox and developmental zone shifts under non-lethal thermal stress. (a–b) Representative confocal images showing root tips under mock conditions (a) or after treatment with 5 nM RGF1 (b) at 22°C and 31°C. Propidium iodide (PI, red) was used to visualize cell outlines, and the reduced-oxidized (RO) index was used to assess relative cellular redox status using the cytosolic GRX-roGFP2. Arrowheads in the PI images indicate the position of the boundary between the meristematic and elongation zones, defined based on cortex cell length. Arrowheads in the RO images indicate the first detectable oxidized cell domain. Scale bars, 100 µm. (c–d) Quantitative analysis of (a) and (b). (c) Distance from the QC to the first detectable oxidized cell domain as an indicator of spatial redox-state shift. (d) Mean RO index values quantified in regions proximal to the oxidized cell domain within the meristematic zone. Different letters indicate significant differences ($p < 0.05$), determined by ART-ANOVA followed by the Games–Howell test in (c), and by Fisher's ANOVA followed by the Tukey–Kramer test in (d). The annotation in the upper left indicates the interaction effect between temperature and RGF1 treatment (***: $p < 0.001$, **: $p < 0.01$, *: $p < 0.05$, ns: non-significant). In the boxplots, each circle represents an individual data point, and sample sizes are indicated below the corresponding boxes. The horizontal line within each box denotes the median, while the upper and lower hinges represent the 75th and 25th percentiles (interquartile range, IQR), respectively. Whiskers extend to 1.5 × IQR.

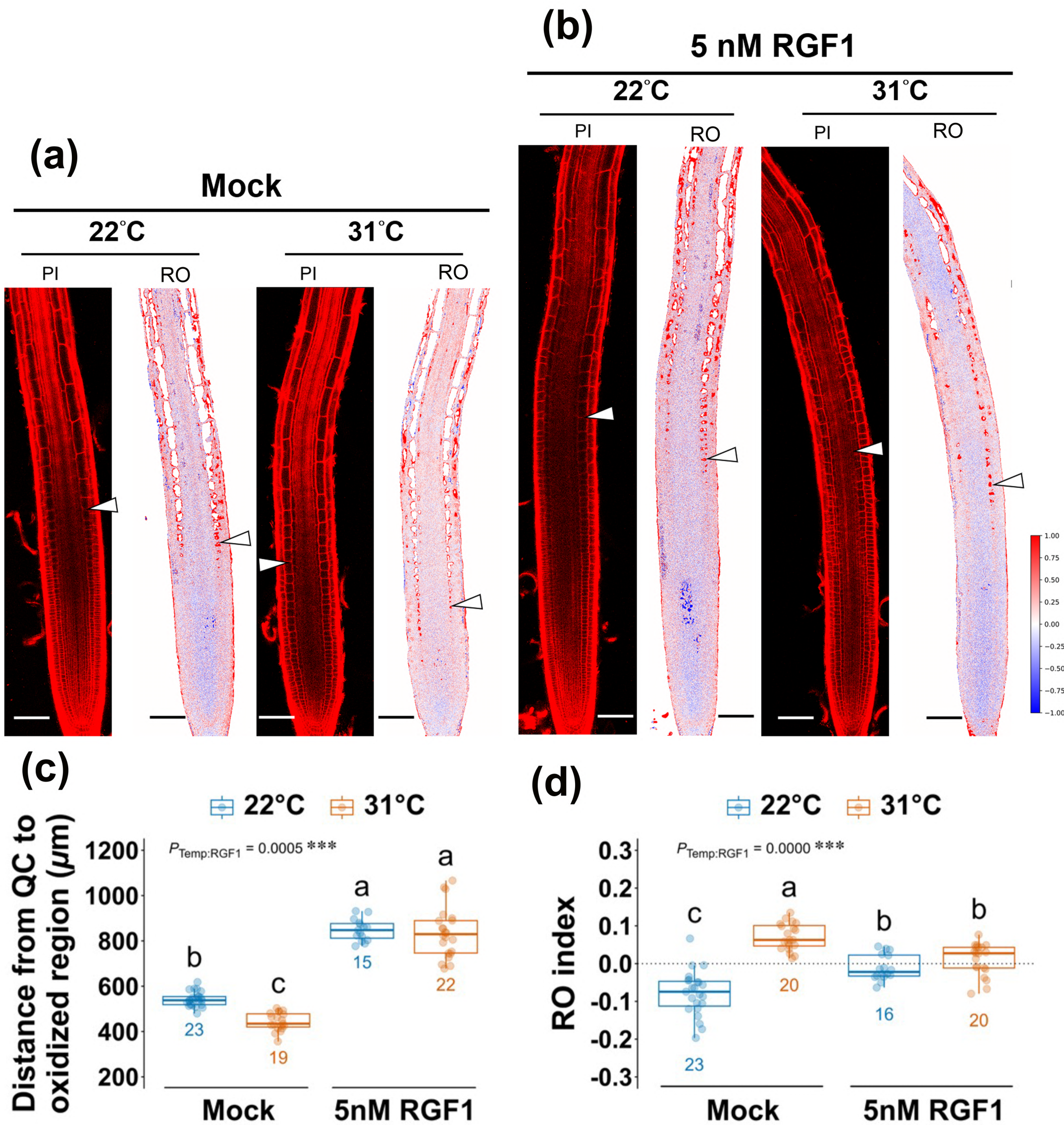

(a)
Mock
22°C
31°C
PI
RO
PI
RO
(b)
5 nM RGF1
22°C
31°C
PI
RO
PI
RO
1.00
0.75
0.50
0.25
0.00
−0.25
−0.50
−0.75
−1.00
(c)
22°C
31°C
Distance from QC to oxidized region (μm)
P_Temp:RGF1 = 0.0005 ***
1200
1000
800
600
400
200
b
c
a
a
23
19
15
22
Mock
5nM RGF1
(d)
22°C
31°C
RO index
P_Temp:RGF1 = 0.0000 ***
0.3
0.2
0.1
0.0
-0.1
-0.2
-0.3
c
a
b
b
23
20
16
20
Mock
5nM RGF1
