# Supplementary material for: RGF signaling bridges root development and nonlethal thermal stress adaptation
Source: New Phytol. 2026 Jul 5;251(5):2656–71. doi: 10.1111/nph.71392 (PMC13443300; doi:10.1111/nph.71392)
Supplement: Supplementary file 2 [file NPH-251-2656-s006.pdf]

Quantifying fluorescent protein sensor cytGRX-roGFP2 redox state with a reduced–oxidized (RO) index

Procedure:

1. Load image

   1.1 Read confocal microscopy CZI file.

   1.2 Extract fluorescence channels:
   - E405: 405 nm excitation channel
   - E488: 488 nm excitation channel
   - PI: Propidium iodide channel

2. Root region segmentation

   For each channel $C \in \{E_{405}, E_{488}\}$:

   2.1 Normalize intensity values of C to the range [0, 255].

   2.2 Apply a median filter.

   2.3 Compute an Otsu threshold T_c.

   2.4 Generate binary mask M_c such that

$$M_c(x,y) = \begin{cases} 1, & C(x,y) > T_c \\ 0, & otherwise \end{cases}$$

   2.5 Retain the masked image $C' = C \cdot M_c$.

3. Protein presence mask

$$M_{protein} = M_{405} \vee M_{488}$$

   where $\vee$ denotes the logical OR operator.

4. Saturated pixel removal

   For each processed channel $C' \in \{E'_{405}, E'_{488}\}$:

   4.1 Extract all non-zero pixels.

   4.2 Compute percentile thresholds

$$T_{high} = P_{99}(C')$$

   4.3 To avoid too many tied values, only when the ratio of unique intensity values exceeds 0.3, set

$$C'(x,y) = 0 \; if \; C'(x,y) > T_{high}$$

5. Redox index calculation

5.1 Square both channels

$$E''_{405} = (E'_{405})^2$$

$$E''_{488} = \left(E'_{488} \cdot \frac{\max(E'_{405})}{\max(E'_{488})}\right)^2$$

5.2 Compute the redox index (RO)

$$RO = \frac{E''_{405} - E''_{488}}{E''_{405} + E''_{488}}$$

5.3 Replace undefined values with zero.

5.4 Restrict analysis to valid protein regions

$$RO = RO \cdot M_{protein}$$

6. Reduced-Oxidized (RO) index classification

$$-1 \leq RO \leq 1$$

- RO > 0 indicates a more oxidized state
- RO < 0 indicates a more reduced state
